# Apicoplast phylogeny reveals the position of *Plasmodium vivax* basal to the Asian primate malaria parasite clade

**DOI:** 10.1038/s41598-019-43831-1

**Published:** 2019-05-13

**Authors:** Nobuko Arisue, Tetsuo Hashimoto, Satoru Kawai, Hajime Honma, Keitaro Kume, Toshihiro Horii

**Affiliations:** 1 Department of Molecular Protozoology, Research Institute for Microbial Diseases, Osaka University, Osaka, 565-0871 Japan; 2 Graduate School of Life and Environmental Sciences, University of Tsukuba, Tsukuba, 305-8572 Japan; 3 Laboratory of Tropical Medicine and Parasitology, Dokkyo Medical University, Tochigi, 321-0293 Japan; 4 Department of International Affairs and Tropical Medicine, Tokyo Women’s Medical University, Tokyo, 162-8666 Japan

**Keywords:** Phylogenetics, Malaria

## Abstract

The malaria parasite species, *Plasmodium vivax* infects not only humans, but also African apes. Human specific *P. vivax* has evolved from a single ancestor that originated from a parasite of African apes. Although previous studies have proposed phylogenetic trees positioning *P. vivax* (the common ancestor of human and African ape *P. vivax*) within the assemblages of Asian primate parasites, its position has not yet been robustly confirmed. We determined nearly complete apicoplast genome sequences from seven Asian primate parasites, *Plasmodium cynomolgi* (strains Ceylonensis and Berok), *P. knowlesi P. fragile*, *P. fieldi*, *P. simiovale, P. hylobati*, *P. inui*, and an African primate parasite, *P. gonderi*, that infects African guenon. Phylogenetic relationships of the *Plasmodium* species were analyzed using newly and previously determined apicoplast genome sequences. Multigene maximum likelihood analysis of 30 protein coding genes did not position *P. vivax* within the Asian primate parasite clade but positioned it basal to the clade, after the branching of an African guenon parasite, *P. gonderi*. The result does not contradict with the emerging notion that *P. vivax* phylogenetically originated from Africa. The result is also supported by phylogenetic analyses performed using massive nuclear genome data of seven primate *Plasmodium* species.

## Introduction

Malaria, one of the most serious infectious diseases, remains a major source of global morbidity and mortality in the tropics and is caused by the genus *Plasmodium*. Malaria parasites comprise a diverse group of over 250 *Plasmodium* species that infect primates, rodents, birds, and reptiles^1^. The ability of *Plasmodium* spp. to adapt to a number of hosts and varying selective pressures emphasizes the need for a better phylogenetic assessment of human malaria parasites and their relatives, as a key issue for the biology of this pathogen and its many ramifications for better understanding of the disease. However, the phylogenetic positions of human and non-human malaria parasites in the *Plasmodium* species tree are not clearly known.

Among the five human malaria parasites, the most virulent one, *P. falciparum*, has been shown to be phylogenetically closely related to the malaria parasites of great apes^2–4^ and to branch early in the tree of *Plasmodium* clade. However, phylogenetic positions of the studied human parasites, *P. malariae* and *P. ovale* have not been resolved for a long time. Multi-gene phylogeny of the apicoplast genome-encoded protein coding genes demonstrated a close relationship between *P. ovale* and rodent *Plasmodium* species. The maximum likelihood (ML) tree of the phylogeny positioned *P. malariae* at the branch between the primate parasite clade and the clade linking rodent parasites with *P. ovale*, but the relationship was not statistically significant^5,6^. Genome analyses of *P. ovale* and *P. malariae* were completed in 2017 and branching positions of these two species were clearly resolved by multigene phylogeny of nuclear genome-encoded genes^7^; the positions were identical with those of the ML tree of apicoplast phylogeny^5,6^.

On the other hand, *P. vivax*, the human parasite most prevalent outside Africa, is believed to have originated from Asian primate parasites including *P. knowlesi* that can infect both human and Asian macaque. Multi-gene phylogeny using two nuclear genes, β-tubulin and cdc2, and an apicoplast gene, tufA revealed that *P. vivax* was nested within the Asian primate parasite clade and an estimated time frame for the origin of the current *P. vivax* populations, the authors concluded that *P. vivax* had originated in Asia^8^. Other phylogenetic analyses also positioned *P. vivax* within the Asian primate parasite clade^9–12^, although no clear resolution was obtained for the exact position of *P. vivax* in these analyses. In addition, a haplotype network, parasite migration patterns, demographic history, and co-phylogeny mapping by Mu *et al*.^13^ supported the Asian origin of *P. vivax* via a host switch from macaque monkeys.

However, parasites of the genus *Plasmodium* that are very closely related to human *P. vivax* were found to infect great apes in Africa, and these were regarded as African great ape *P. vivax*^14–16^. Phylogenetic analyses of the mitochondrial, apicoplast, and six nuclear genome-encoded genes revealed that *P. vivax* lineages that are specific to humans are monophyletic within the African great ape *P. vivax* lineages^16^. Two genome and 9 draft genome sequences of chimpanzee *P. vivax* (*P. vivax*-like) were reported recently and genome-wide phylogenetic analyses revealed that these chimpanzee *P. vivax* form a genetically distinct clade from human *P. vivax*^17^. Furthermore, a new species, *Plasmodium carteri*, which is closely related to the ape and human *P. vivax* clade was found in wild chimpanzee^18,19^. These reports indicated that human *P. vivax* had originated in Africa from great ape *P. vivax* parasites and ape parasites also originated in Africa. However, the branching position of *P. vivax*, including both the human and ape parasites in the tree of the genus *Plasmodium*, has not clearly been revealed, because taxon sampling of the trees in the above reports were sometimes sparse lacking major *Plasmodium* lineages except for Asian primate *Plasmodium* species. Therefore, whether *P. vivax* is nested within the Asian primate parasite clade need to be further investigated. Since an African guenon parasite, *P. gonderi*, is important to infer phylogenetic relationships between *P. vivax* and Asian primate parasites, we here included the data from *P. gonderi* and examined multi-gene phylogeny using apicoplast genome-encoded and nuclear genome-encoded genes.

Apicoplast is a plastid-derived organelle lacking photosynthetic ability. It is widely found in apicomplexan parasites^20,21^ and possesses a 35 kb circular genome, which encodes translation and transcription related protein genes, rRNAs, tRNAs, and several other genes including those with unknown functions^22^. Sequences of the apicoplast genome-encoded genes have many advantages in the phylogenetic inference of the inter-species relationships among the genus *Plasmodium*^5,6^. Although apicoplast genomes of *Plasmodium* parasites generally show extremely high A + T contents, these are almost constant between species, and thus the possibility of the misleading inference stemming from extreme compositional heterogeneity^23–25^ could be ruled out in the phylogeny of apicoplast genome-encoded genes. All of the 30 protein coding genes are a single gene, enabling orthologous comparison to infer organismal phylogeny. Sequences of the genes are appropriately diverged for the inference of the relationships within the clade of the genus *Plasmodium* and can be reliably aligned without ambiguity. In addition, since more phylogenetic information is included in the apicoplast genome (35 kbp) than in the mitochondrial genome (6 kbp), apicoplast genome-based phylogeny could resolve the relationship between *Plasmodium* species more precisely than mitochondrial genome-based phylogeny that has been widely used for the phylogenetic analysis of malaria parasites^10,26–29^. For these reasons, apicoplast genome sequences are considered to be more suitable than those of the mitochondria for phylogenetic studies of malaria parasites.

In this study, we determined nearly complete apicoplast genome sequences from eight Asian primate *Plasmodium* species and strains: *P. cynomorgi* strains (Ceylonensis and Berok), *P. knowlesi*, *P. fragile*, *P. fieldi*, *P. semiovale, P. hylobati*, and *P. inui*, and an African guenon parasite, *P. gonderi*. An evolutionary relationship of the genus *Plasmodium* clade was analyzed using 30 apicoplast genome-encoded protein genes. DNA and protein-based analyses consistently revealed that the tree did not nest *P. vivax* within the Asian primate malaria parasite clade, but positioned it basal to the clade, after the branching of an African guenon parasite, *P. gonderi*. The result suggests that *P. vivax* could phylogenetically have an African origin. The result is also supported by the phylogenetic analysis using 627 nuclear genome-encoded genes from seven *Plasmodium* species.

## Results

### Gene repertory, arrangement, and features of the apicoplast genomes of *Plasmodium* spp

We determined nearly complete nucleotide sequences of the apicoplast genome from nine *Plasmodium* species and strains (Supplementary Table S1). A small region around tRNA-Ile in the inverted repeats (IRs) could not be determined because of technical difficulties. Gene repertory and gene arrangement of each apicoplast genome were almost identical to those reported for several *Plasmodium* species^5,6,22^. All the *Plasmodium* apicoplast genomes contained genes for small subunit (SSU) and large subunit (LSU) rRNAs, 25 species of tRNAs, 17 ribosomal proteins, 3 subunits of RNA polymerase, elongation factor Tu (tufA), caseinolytic protease C (clpC), sulfur mobilizing protein B (sufB), and 7 open reading frames of unknown function (Supplementary Table S2), all packed in the genome tightly with short intergenic regions. Compared to the mitochondrial genomes of *Plasmodium* species with around 70% A + T content^10^, apicoplast genomes sequenced in this study showed high A + T content, ranging from 86.5% (*P. knowlesi*) to 87.1% (*P. inui*), consistent with the average A + T richness of previously reported nine *Plasmodium* species (average 86.7%)^5^.

### *Plasmodium* phylogeny based on the dataset of 30 protein coding genes

Due to high sequence similarity (93.5–99.8%) (Supplementary Table S3), sequences of both rRNA and tRNA genes did not possess sufficient phylogenetic signals to resolve relationships among *Plasmodium* lineages. We, therefore, focused on the 30 protein-coding genes, whose sequence similarity was relatively low, 72.0–94.9% at the nucleotide level (Supplementary Table S3), none of which were duplicated.

Nucleotide and amino acid compositions of the datasets used for the combined phylogeny of 30 protein coding genes are summarized in Supplementary Fig. S1. Akin to the A + T contents of the whole genome in the *Plasmodium* species described above, the present DNA data set of 30 genes were extremely A + T-rich. In the first and second codon position, a few guanines and cytosines are observed, but in the third codon position, more than 96% were A + T, and cytosine occupied less than 1%. Because of the A + T richness of genes, amino acids encoded by A + T-rich codons, such as, asparagine, isoleucine, leucine, lysine and tyrosine showed high composition rates. However, no extreme compositional bias between species was observed both in the DNA and protein data sets, demonstrating that the present phylogenetic inference may not have been artificially affected by compositional heterogeneity across lineages^30^

**Figure 1:**
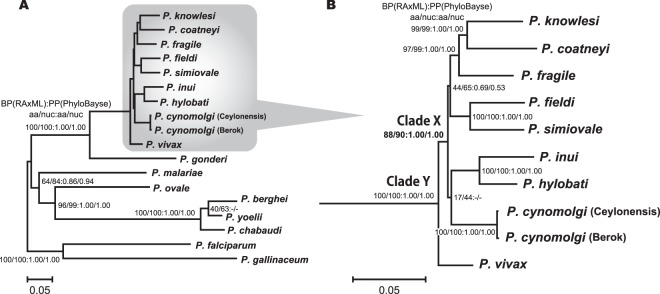
Maximum likelihood tree of *Plasmodium* species. Unambiguously aligned positions of 30 protein coding genes were concatenated, and the resulting 6,937 amino acid and 20,811 nucleotide positions were used for the tree inference. RAxML 7.2.8 program^31^ with GTR +Γ model was used for the both amino acid and DNA datasets. For DNA data set, 3 codon positions were partitioned and applied for the program. Tree portion highlighted in (**A**) was enlarged and shown in (**B**). Bootstrap analyses were performed for 1000 replicates, and bootstrap values are shown for each internal branch with probabilities assessed by PhyloBayes^32^.

Maximum likelihood (ML) trees of RAxML^31^ analyses using the DNA and protein data sets for 30 protein coding genes were identical in their topologies. As a representative figure, the tree of DNA data set based on GTR +Γ model with a partition for three codon positions is shown in Fig. 1. Bootstrap proportion (BP) and posterior probability (PP) values inferred by the ML and PhyloBayes^32^ analyses are shown on the internal branches of the tree. Asian primate *Plasmodium* species were monophyletic and the corresponding clade (clade X in Fig. 1B) was supported with high BP and PP values either in the analyses of DNA or protein data sets. *P. vivax* was positioned at the base of the Asian primate parasite clade, after the divergence of an African guenon parasite, *P. gonderi*. Both the monophyly of *P. vivax* with Asian primate parasites (clade Y in Fig. 1B) and the sister group relationship of *P. gonderi* to the clade Y were highly supported in all analyses examined, demonstrating that the branching position of *P. vivax* is almost completely resolved and thus provides a plausible explanation for positioning *P. vivax* basal to the Asian primate parasites clades.

### Robustness of the phylogenetic inference under different evolutionary models

To exclude the possibility that the supported clade that groups Asian primate parasites, excluding *P. vivax* (reconstructed in Fig. 1), was an artifact related to model misspecification, we analyzed both DNA and protein datasets under a variety of substitution models.

**Figure 2:**
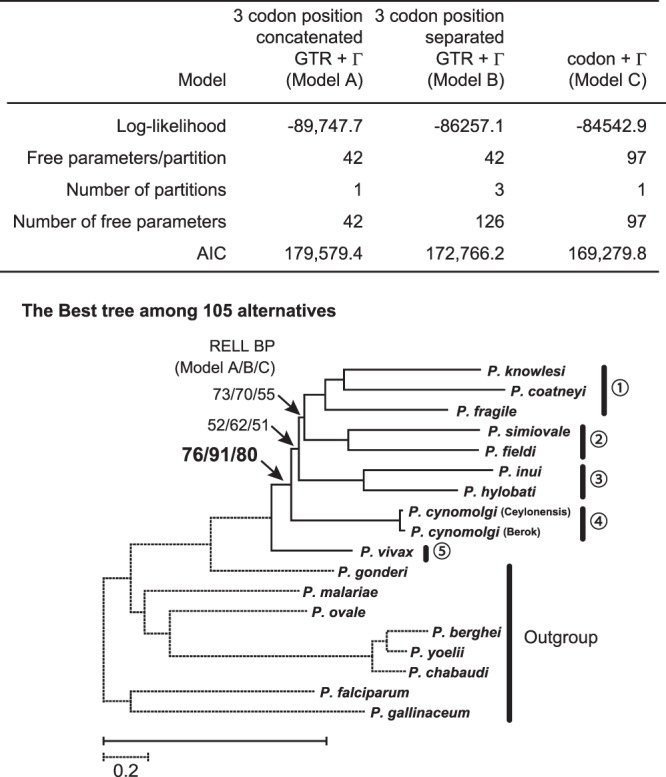
Model test for phylogeny. Eighteen *Plasmodium* species were classified into 5 groups and an out group, and exhaustive analyses of the 105 trees with three nucleotide substitution model (Model A to C) were applied for the analyses by using PAML 4.8^33^. Akaike Information criterion (AIC) values were calculated to evaluate the most appropriate model among the three. RELL bootstrap probabilities were shown for internal branches of the tree inferred by the codon +Γ model (Model C).

In the analyses of the DNA data set, to compare a codon substitution model implemented in PAML program^33^ with GTR +Γ model with or without partition for three codon positions, we exhaustively compared 105 possible trees, assuming six constrained lineages in advance as described in the Materials and Methods section. All the three models examined (Models A, B, and C) favored the same tree topology shown in Fig. 1 as the ML tree among 105 alternative trees analyzed (Fig. 2). Comparison of the Akaike’s information criterion [AIC]^34^ values for the three models revealed that the codon +Γ model (Model C) with the lowest AIC value was the best, and far better approximated the data set than the GTR +Γ model (Model A or Model B). Compared to the concatenate model for three codon positions (Model A), the partition model (Model B) improved the model approximation, and thus was considered to be more appropriate. On the other hand, approximately unbiased (AU) test comparing the 105 possible trees among the six *Plasmodium* groups with 18 OTUs did not necessarily exclude all trees in which *P. vivax* is nested within the assemblages of Asian primate parasites in the analysis using the codon +Γ model (Model A) (data not shown). However, monophyly of the Asian primate parasites, excluding *P. vivax*, was supported (80%) by resampling estimated log likelihood (RELL) BP value^35^ (Fig. 2).

**Figure 3:**
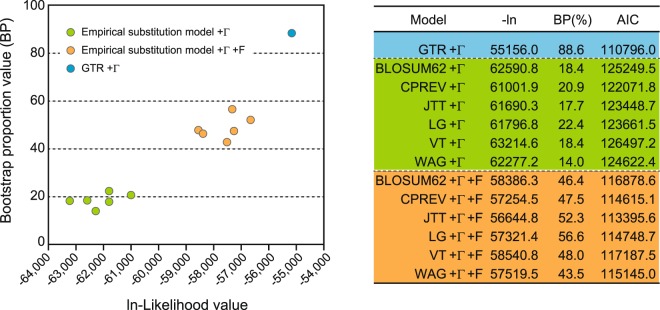
Impact of the substitution model for bootstrap proportion value and the maximum likelihood value of phylogenetic tree. Maximum likelihood trees were inferred using RAxML 7.2.8 program^31^ with several amino acid substitution models and 6,937 amino acid positions. Maximum likelihood value (-ln) and bootstrap probability value (BP) on the internal branch, which shows clade *P. vivax* with Asian primate malaria parasites, were plotted for each analysis, and Akaike Information criterion (AIC) values were calculated to evaluate the most appropriate model.

In the protein data set, in order to compare the result of the analysis with GTR +Γ model with those of several empirical amino acid substitution models as described in the Materials and Methods section, RAxML analyses using BLOSUM62 +Γ, CPREV +Γ, JTT +Γ, LG +Γ, VT +Γ and WAG +Γ models were performed with or without ‘F’ option. Although all the analyses supported the clade Y in Fig. 1B and the close affinity of *P. gonderi* with the clade Y, difference of the empirical models demonstrated a large impact on the log-likelihood value of the ML tree and the nodal support value for the monophyly of Asian primate parasites, excluding *P. vivax* (clade X in Fig. 1B). Comparison of the AIC values for all the models examined revealed that GTR +Γ is the best, with far smaller AIC value, among all the models (Fig. 3). Introduction of ‘F’ option into the empirical models remarkably improved the AIC values, but these values were not comparable with the AIC value of the GTR +Γ model with many parameters. The less the AIC value of the model becomes, or the more appropriate the model is, the BP value for a clade X increases, suggesting that the monophyly of Asian primate parasites excluding *P. vivax* is likely to occur. Since the codon +Γ model for the DNA dataset and the GTR +Γ model for the protein data set, both of which are considered to be the best models, consistently supported the monophyly of Asian primate parasites, positioning *P. vivax* at the base of Asian primate parasites in the phylogeny of apicoplast genome-encoded genes seems to be accurate.

### *Plasmodium* phylogeny based on the nuclear genome-encoded 627 genes

**Figure 4:**
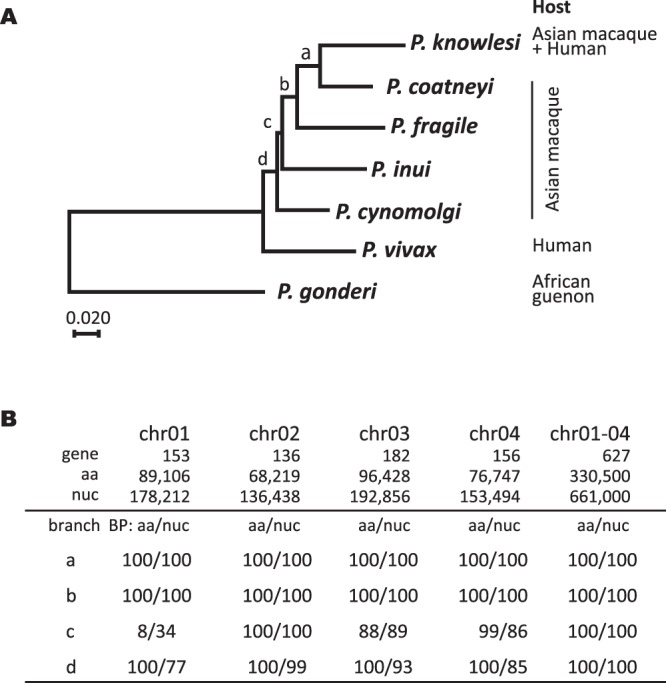
Tree inference of seven *Plasmodium* species using nuclear genome encoded genes orthologous to genes on *P vivax* chromosome 1 to chromosome 4. (**A**) Maximum Likelihood tree. Unambiguously aligned positions from 627 protein coding genes were concatenated and the resulting 330,500 amino acid and the first and second codon positions of 661,000 nucleotide positions were used for the tree inference. RAxML 7.2.8 program^31^ with GTR +Γ model was used for both the amino acid and DNA datasets. For DNA data set, the first and second codon positions were partitioned and applied for the program. (**B**) Bootstrap probability value (BP) on the internal branches, a to d, shown in (**A**). Bootstrap analysis was performed for 100 and 1000 replicates for amino acid and DNA dataset, respectively.

Phylogenetic relationship malaria parasites were analyzed on the basis of nuclear genome-encoded 627 genes from *P. vivax* chromosomes 1 through 4 and their orthologous genes from five Asian primate malaria parasites [991,500 nucleotide and 330,500 amino acid positions] (Supplementary Table S4) using an African guenon parasite, *P. gonderi*, as an outgroup. A + T content of the nuclear genome varied across seven species more remarkably than that of apicoplast genome (Fig. S2). Since the largest variation of the A + T content was observed in the third codon position, the position was removed from DNA dataset and the first and second codon positions were used for the analysis of DNA data set. The ML tree positioned *P. vivax* basal to the Asian primate parasites with 100% BP support value in the analyses of both protein and DNA datasets (Fig. 4). When we analyzed each gene groups orthologous to genes on *P. vivax* chromosome 1 to 4 separately, the ML tree was the same as the ML tree of the whole data set as shown in Fig. 4 for the gene groups orthologous to genes on *P. vivax* chromosomes 2, 3, and 4, whereas in the ML tree of gene group orthologous to genes on *P. vivax* chromosome 1 (267,318 nucleotide and 89,106 amino acid positions), the branching position of *P. vivax* next to *P. gonderi* in the ML tree as demonstrated in Fig. 4 was reconstructed, but the branching position of *P. cynomolgi* and *P. inui* were different from the ML tree. On the other hand, when we applied various substitution models as shown in Supplementary Table S5 for the whole datasets, BP values of all branches were consistently supported by 100% without model dependency. AU test applied to the possible 15 tree topologies for the 5 lineages rejected the possibility that the position of *P. vivax* nested within the Asian primate malaria parasite clade significantly (*p* < 0.05) (Supplementary Table S6). Although these analyses use only chromosomes 1 through 4 and lack other Asian primate taxa, *P. hylobati, P. fieldi* and *P. simiovale*, the branching position of *P. vivax* next to African guenon parasite of *P. gonderi*, at the base of the Asian primate *Plasmodium* group, is consistent with the position in the ML tree of the apicoplast genome-encoded genes (Figs 1, 2) and supported the result of the present apicoplast phylogeny.

## Discussion

More than three decades ago *P. vivax* was believed to be of an African origin because of the high rate of Duffy-negative phenotype in African people^36^. *P. vivax* uses the Duffy antigen receptor for chemokines (DARC) to invade human red blood cell^37^. As the Duffy-negative phenotype is resistant to *P. vivax* invasion, this mutation was supposed to be selected in the *P. vivax* in endemic areas such as West Africa. Recently, susceptibility of *P. vivax* in African wild-living apes such as chimpanzee, gorilla and bonobo^3,4,14–16^ was demonstrated, while resistance of *P. vivax* in Asian macaque^38^ was also shown. These data suggest the African origin of *P. vivax*.

However, previous phylogenetic analyses using mitochondrial genome-encoded genes, apicoplast genome-encoded genes or some nuclear genes consistently positioned *P. vivax* nested within the clade consisting of Asian primate parasite species^8–12^. If the phylogenetic position of *P. vivax* revealed by these analyses were true, then the phylogeny would support the hypothesis that *P. vivax* originated in Asia due to the host switch from macaque to human, which essentially has long been accepted in the field of *Plasmodium* phylogeny^8,12,13^. However, most of these analyses did not resolve the position of *P. vivax* with high BP or PP support values.

On the other hand, recent discoveries of African ape *P. vivax* (*P. vivax*-like) isolates and a new species, *P. carteri*, which is closely related to the human and ape *P. vivax* demonstrated that *P, vivax* is an African origin^14–19^. Phylogenetic trees in these reports that include various isolates/strains of *P. vivax* (ape and human) and *P. carteri* consistently revealed that all of these isolates/strains were monophyletic excluding the Asian primate *Plasmodium* species^16–19^. Moreover, human *P. vivax* isolates/strains formed a clade in the assemblages of the African ape *P. vivax* isolates^16–19^. These results strongly suggested that none of the extant human *P. vivax* were of Asian macaque origin but were of African ape origin.

The uncertainty of the branching position of *P vivax* in the previous molecular phylogeny was caused by insufficient sequence data both in the number of genes for multi-gene phylogeny and of the taxa related to *P. vivax*. As shown in Fig. 1, the lengths of the internal branches for the subtree of *P. vivax* and Asian primate parasites are very short, suggesting that these species diverged within a short time period. In such a case, to resolve the relationships between these closely related species, large amount of sequence data are necessary for phylogenetic analysis. However, the data used in the previous phylogenetic analyses have always been inadequate, and thus the trees inferred by these analyses have not resolved the above relationships clearly. In our present analyses using 30 apicoplast genome-encoded genes from 18 *Plasmodium* species, the best model with the least AIC value, either in protein phylogeny or in DNA phylogeny, revealed that *P. vivax* is positioned at the base of the clade including all Asian primate *Plasmodium* species by means of highly supportive data, and the *P. vivax* branch is next to the divergence of African guenon parasite, *P. gonderi*. The results from the apicoplast genome-encoded genes were also supported by the analyses using 627 nuclear genes from seven *Plasmodium* species. Thus, in addition to the finding that African apes are natural hosts of *P. vivax*, the branching position of *P. vivax*, next to *P. gonderi* and before the common ancestor of Asian primate parasites supports an African origin of *P. vivax* in the tree of the genus *Plasmodium*. *P. vivax* (and plausibly also *P. carteri*) most likely diverged from the ancestral species that was closely related to ancestral *P. gonderi* in Africa. The importance of our present results is that *P. vivax* was at the first time phylogenetically linked to an African guenon parasite, *P. gonderi*. The evolutionary position of *P. vivax* implies that *P. vivax* originated in Africa, and the Asian macaque *Plasmodium* parasites likely originated from ancestral lineage(s) of *P. vivax* or its closely related *species* due to the host switch between African apes/archaic humans and Asian macaques.

To further resolve the evolutionary history and host switch events of *Plasmodium* species more clearly, phylogenetic studies using massive nuclear genome sequences with adequate taxon sampling is necessary.

## Materials and Methods

### Genomic DNA (gDNA) preparation

*Plasmodium* species and strains used for this study are listed in Table. S1 (Supplementary Information). Most of the parasites were obtained from the American Type Culture Collection (ATCC), unless described previously^26,39^. gDNA of *Plasmodium* species were extracted from parasitized red blood cells using QIAamp DNA Blood Mini Kit (QIAGEN) according for manufacturer’s instructions.

### DNA sequencing

To determine the nucleotide sequence of the apicoplast genome, the putative genome sequence was divided into several overlapping regions and each region was amplified through a polymerase chain reaction (PCR) using specific primers. Primer sequences and their corresponding regions are listed in Fig. S3 (Supplementary Information). Amplification was carried out using DNA polymerases: KOD-FX or KOD-FX-Neo (TOYOBO) with cycle conditions: 94 °C for 2 min; 40 cycles at 94 °C for 30 sec, 59 °C for 30 sec, 68 °C for 1 to 6 min (1 min for 1 kb amplicon length). PCR conditions were optimized for each reaction. Amplified products were purified using the QIAquick PCR Purification Kit (QIAGEN) and directly sequenced using the 3130 Genetic Analyzer (Applied Biosystems) and Big Dye Terminator Cycle Sequence kit v3.1 (Applied Biosystems). Both the strands of each DNA fragment were sequenced by primer walking. The draft sequence of *P. cynomolgi* (Berok) apicoplast genome was kindly given by Jane M Carlton. We corrected the sequence by Sanger method and used for the analysis. Apicoplast genome sequences reported in this study have been deposited in DDBJ/EMBL/GenBank with accession numbers **AP018101–AP018109**.

### Sequence alignment and phylogenetic analyses

Nucleotide and predicted amino acid sequences of 30 protein coding genes of the apicoplast genome were aligned using ClustalW ver. 2.1^40^ with manual corrections using the alignment editor packaged in GENETYX ver. 11 (GENETYX). Unambiguously aligned positions were selected from the 30 gene alignments, and the concatenated DNA and protein data sets with 20,811 nucleotide and 6,937 amino acid positions were subjected to phylogenetic analyses shown in Fig. 1. The maximum likelihood (ML) method based on RAxML ver. 7.2.8 program^31^ was used for inferring the ML tree. Models assumed for transition probability were GTR models^41,42^ with among-site rate heterogeneity approximated by discrete Γ distribution with four categories^43^ (GTR +Γ model) for both nucleotide and amino acid substitution processes. Empirical amino acid substitution models, BLOSUM62 +Γ, CPREV +Γ, JTT +Γ, LG +Γ, VT +Γ, and WAG +Γ were also applied to the protein data set with or without the “+F” option which uses observed amino acid frequencies of the protein data set for calculation of ML. Partition models for 30 genes were not assumed for both the DNA and protein analyses, because these parameter-rich models were not necessarily more appropriate than concatenate models in the previous analyses of the apicoplast genome-encoded gene data set^5,11^. However, in the DNA analysis, a partition model for three codon positions^44^ was also examined in addition to a concatenate model. In each RAxML analysis, ten maximum parsimony trees were used as initial trees to heuristically search for an optimal ML tree, whereas 1,000 bootstrap replicates were analyzed for calculating bootstrap proportion (BP) values. In addition, PhyloBayes ver 4.1c^32^ was used for Bayesian inference using the CAT + GTR +Γ4 model, applied to both the DNA and protein datasets. Two independent Markov Chain Monte Carlo (MCMC) chains were run for 23,592 and 4,658 generations, respectively, for DNA and protein datasets. The burn-in period was settled at 5,000 for the DNA and 1,000 for the protein datasets, and these generations were removed. Maxdiff values were 0.0285 and 0.0802 in the DNA and protein analyses, respectively.

To evaluate the robustness of the inference in the above heuristic analyses of the DNA data set, alternative analyses were done based on the exhaustive search by introducing constraints on several *Plasmodium* groups in advance. The above heuristic analyses consistently and clearly revealed monophyly of *P. knowlesi*, *P. coatneyi*, and *P. fragile* with the former two as a sister group; close relationships were established between *P. fieldi* and *P. simiovale*, between *P. inui* and *P. hylobati*, and between the two isolates of *P. cynomolgi* (Fig. 1). Therefore, we put constraints on these relationships in advance and focused only on the relationships among five *Plasmodium* lineages: (1) [*P. fragile*, (*P. knowlesi*, *P. coatneyi*)], (2) (*P. fieldi*, *P. simiovale*), (3) (*P. inui*, *P. hylobati*), (4) two *P. cynomolgi* isolates, and (5) *P. vivax*. In order to resolve the root of tree for these five lineages, other *Plasmodium* lineages with the relationship shown in the ML tree in Fig. 1A was used as outgroups. Three models, the concatenate (Model A), the partition (Model B) models for three codon positions, and a codon substitution model [Model C]^45^ were assumed in the exhaustive analyses by using PAML program^33^. In the analysis of the codon substitution model (Model C), Miyata’s distance among 20 amino acids^46^ was used with geometric formulae. In these analyses by PAML program, RELL bootstrap values [RELLBP]^35^ were calculated and used as support values for internal branches.

In addition, in order to confirm the results of the analyses for apicoplast genome-encoded genes, we also analyzed a large dataset of nuclear genes. A total of 627 genes from *P. vivax* chromosome 1 through chromosome 4 (Supplementary Table S4) and their orthologous genes from 5 macaque *Plasmodium* species, *P. cynomolgi*, *P knowlesi*, *P. coatneyi*, *P. inui*, and *P. fragile* were obtained from PlasmoDB (http://plasmodb.org/plasmo/). The genes of *P. gonderi* were obtained from public database with accession numbers mentioned in Honma *et al*.^47^. For the alignment of each gene, unambiguously aligned positions were selected, and the concatenated first and second codon positions of DNA (661,000 nucleotide positions) and protein (330,500 amino acid positions) datasets were subjected for phylogenetic analyses based on the ML method. The analyses were done by almost the same methods as those used for the analyses of apicoplast genome-encoded genes.

In order to compare the goodness of different statistical models with different number of parameters, we used Akaike’s Information Criterion (AIC), that is, AIC = − 2 × (log-likelihood) + 2 × [number of free parameters]^34^. The model that minimizes the AIC value is considered to be the most appropriate one among other alternatives. To evaluate the significance of inferred ML tree topologies, 7 *Plasmodium* species were divided for the 5 lineages, (1) *P. coatneyi*, *P. knowlesi* and *P. fragile*, (2) *P. inui*, (3) *P. cynomolgi*, (4) *P. vivax*, (5) *P. gonderi* (outgroup), and AU test was applied to the possible 15 tree topologies using CONSEL^48^.

## Supplementary information

Supplementary Information
