# Supplementary material for: Apicoplast phylogeny reveals the position of Plasmodium vivax basal to the Asian primate malaria parasite clade
Source: Sci Rep. 2019 May 13;9:7274. doi: 10.1038/s41598-019-43831-1 (PMC6514274; doi:10.1038/s41598-019-43831-1)
Supplement: Supplementary file 1 — Supplementary Information [file 41598_2019_43831_MOESM1_ESM.pdf]

Supplementary Information

Apicoplast phylogeny reveals the position of *Plasmodium vivax* basal to the Asian primate malaria parasite clade

Nobuko Arisue[1]*, Tetsuo Hashimoto[2], Satoru Kawai[3], Hajime Honma[4], Keitaro Kume[2] and Toshihiro Horii[1]

1 Department of Molecular Protozoology, Research Institute for Microbial Diseases, Osaka University, Suita, Osaka 565-0871, Japan;

2 Graduate School of Life and Environmental Sciences, University of Tsukuba, Tsukuba 305-8572, Japan;

3 Laboratory of Tropical Medicine and Parasitology, Dokkyo University School of Medicine, Tochigi 321-0293, Japan

4 Department of International Affairs and Tropical Medicine, Tokyo Women's Medical University, Tokyo 162-8666, Japan.

Supplementary Fig. S1 Nucleotide/amino acid compositions of the 30 protein coding genes of the 18 *Plasmodium* apicoplast genome. Positions of 6,937 amino acids and 20,811 nucleotides were used for the analysis. Pco: *P. coatneyi*, Pkn: *P. knowlesi*, Pfr: *P. fragile*, Pfi: *P. fieldi*, Pso: *P. simiovale*, Phy: *P. hylobati*, Pin: *P. inui*, Pcy1: *P. cynomolgi* (ceylonensis), Pcy2: *P. cynomolgi* (Berok), Pvi: *P. vivax*, Pgo: *P. gonderi*, Pma: *P. malariae*, Pov: *P. ovale*, Pbe: *P. berghei*, Pyo: *P. yoelii*, Pch: *P. chabaudi*, Pfa: *P. falciparum*, Pga: *P. gallinaceum*.

Supplementary Fig. S2 Nucleotide/amino acid compositions of the nuclear genome encoded 627 protein genes. 330,500 amino acid and first and second codon position of 661,000 nucleotide positions of the seven *Plasmodium* species were used for the analyses. Pco: *P. coatneyi*, Pkn: *P. knowlesi*, Pfr: *P. fragile*, Pin: *P. inui*, Pcy: *P. cynomolgi*, Pvi: *P. vivax*, Pgo: *P. gonderi*,

Supplementary Fig. S3 PCR primer sequences and amplified *Plasmodium* apicoplast genome regions.

Supplementary Table S1 *Plasmodium* species and accession numbers used in this study.

Supplementary Table S2 Gene component and gene length of apicoplast genome in nine *Plasmodium* species that were newly determined in this study.

Supplementary Table S3 Sequence similarities of *Plasmodium* apicoplast genome-encoded genes.

Supplementary Table S4 Gene ID of *Plasmodium vivax*. These genes and their orthologs of six *Plasmodium* species, *P. coatneyi*, *P. knowlesi*, *P. fragile*, *P. inui*, *P. cynomolgi* and *P. gonderi* were used for the phylogenetic analysis.

Supplementary Table S5 Model test for phylogeny using genome encoded gene sequences of seven *Plasmodium* species

Supplementary Table S6 Approximately unbiased test among 15 tree topologies and several amino acid substitution models.

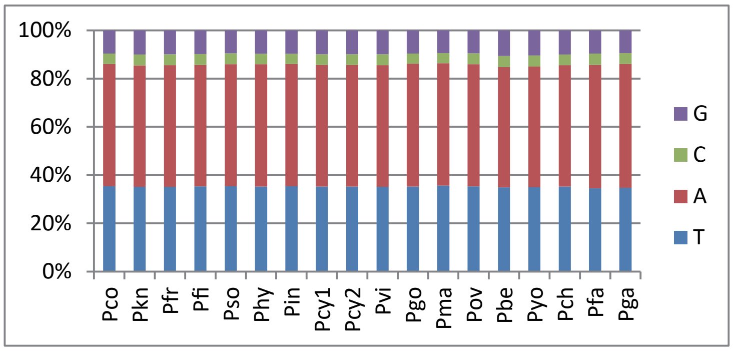

Nucleotide composition (codon-1st)
(AT=84.8 ～ 86.4%)

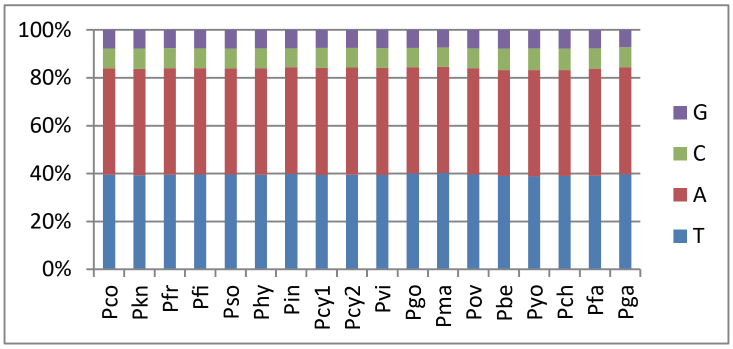

Nucleotide composition (codon-2nd)
(AT=83.2 ～ 84.7%)

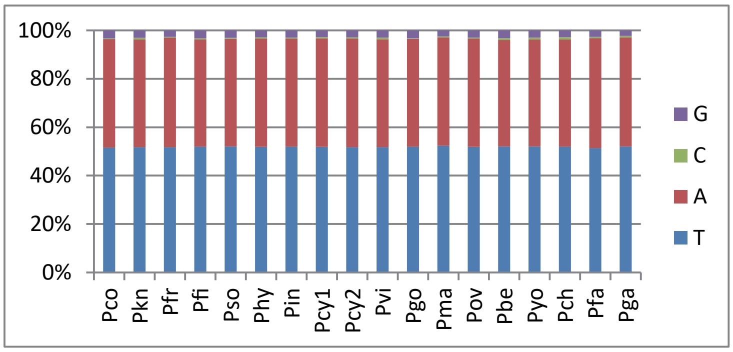

Nucleotide composition (codon-3rd)
(AT=96.0 ～ 97.2%)

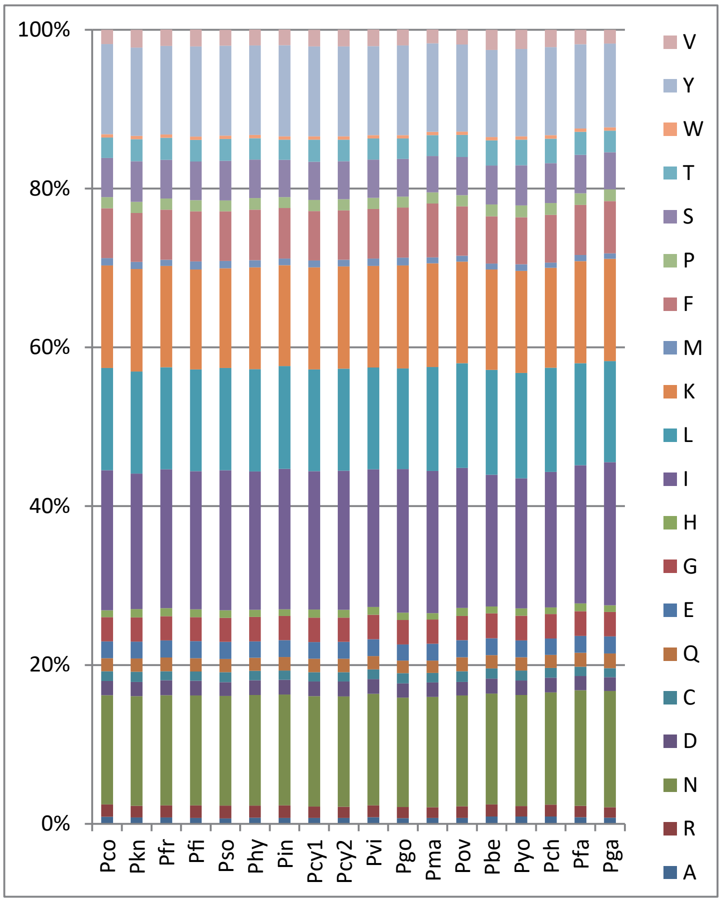

Aminoacid composition

Supplementary Fig. S1 Nucleotide/amino acid compositions of the 30 protein coding genes of the 18 *Plasmodium* apicoplast genome. Positions of 6,937 amino acids and 20,811 nucleotides were used for the analysis. Pco: *P. coatneyi*, Pkn: *P. knowlesi*, Pfr: *P. fragile*, Pfi: *P. fieldi*, Pso: *P. simiovale*, Phy: *P. hylobati*, Pin: *P. inui*, Pcy1: *P. cynomolgi* (ceylonensis), Pcy2: *P. cynomolgi* (Berok), Pvi: *P. vivax*, Pgo: *P. gonderi*, Pma: *P. malariae*, Pov: *P. ovale*, Pbe: *P. berghei*, Pyo: *P. yoelii*, Pch: *P. chabaudi*, Pfa: *P. falciparum*, Pga: *P. gallinaceum*.

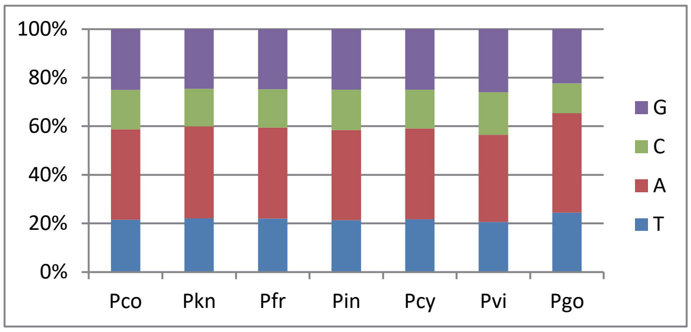

Nucleotide composition (codon-1st)
(AT=56.5 ～ 65.4%)

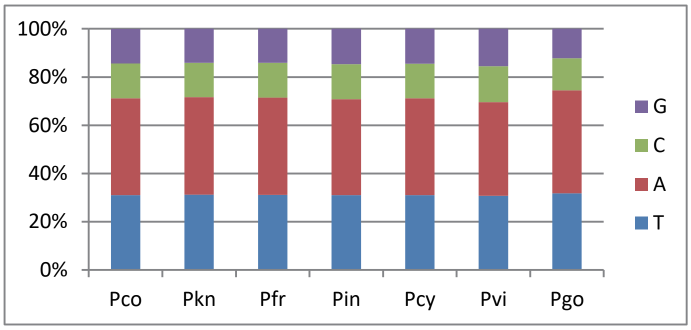

Nucleotide composition (codon-2nd)
(AT=69.6 ～ 74.5%)

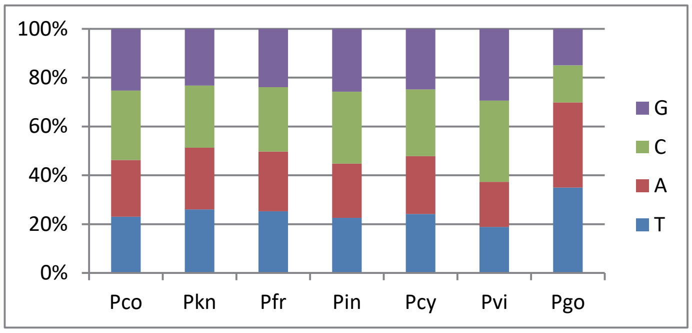

Nucleotide composition (codon-3rd)
(AT=37.4 ～ 69.9%)

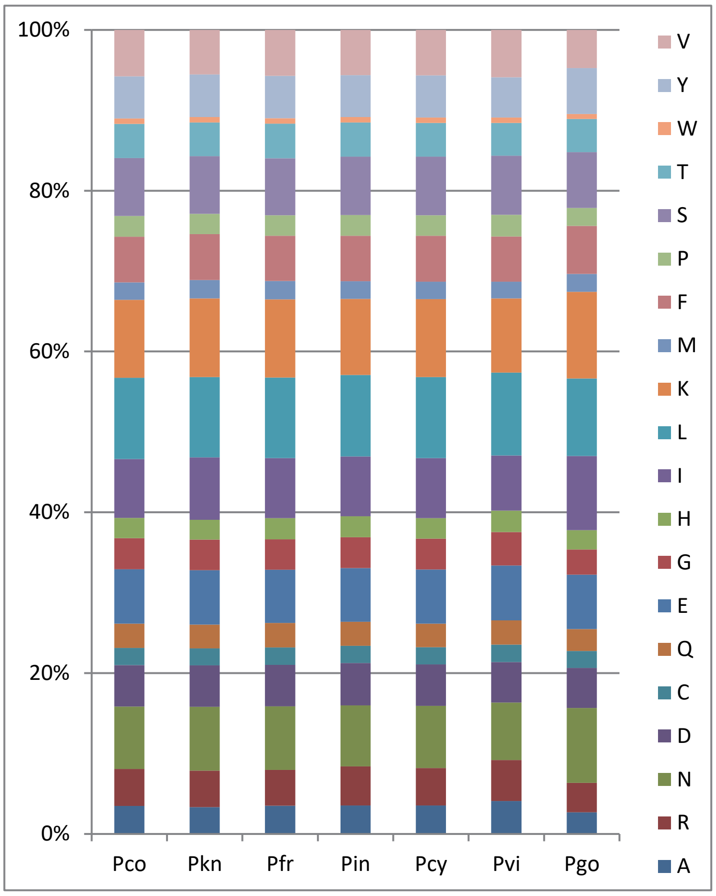

Aminoacid composition

Supplementary Fig. S2 Nucleotide/amino acid compositions of the nuclear genome encoded 627 protein genes. 330,500 amino acid and first and second codon position of 661,000 nucleotide positions of the seven *Plasmodium* species were used for the analyses. Pco: *P. coatneyi*, Pkn: *P. knowlesi*, Pfr: *P. fragile*, Pin: *P. inui*, Pcy: *P. cynomolgi*, Pvi: *P. vivax*, Pgo: *P. gonderi*.

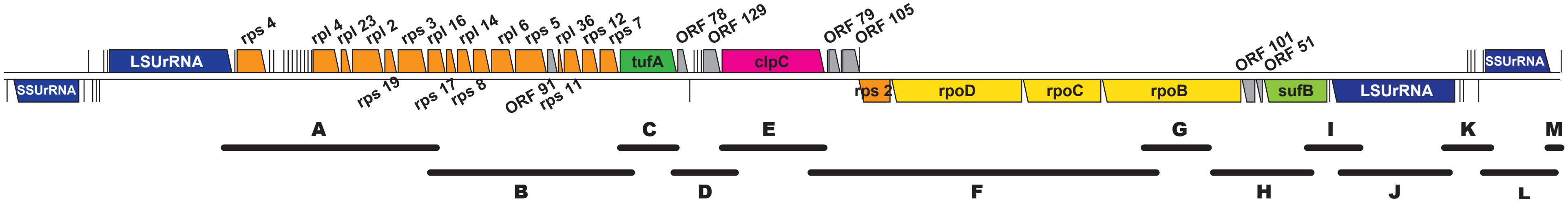

Amprified fragment	primer pairs for 1st PCR		primer pairs for nested PCR	
A	Api_lsu-F1	GTCTGTTCGCCTATTAAAGCGATACGTGAGCT	Api_lsu-F2	CGTCGTGAGACAGTTCGGTCCATATCTAATATAAGT
	Api_rpl16-R1	CTATAGGTCCTTTACCAGCTCCCATTCTAGTTTTTAAAGA	Api_rpl16-R2	CCCATTCTAGTTTTTAAAGACTTTTTAGTTAAAGATTT
B	Api_rpl16-F1	RTATTGGGGTATAATTTCWTTAAATTCWGGDTTTATTACA	Api_rpl16-F2	CWGGDTTTATTACADADAATCMATTRGAARCTTCYARRTT
	Api_tufA-R1	CWGMATGACCDGGACAATCWATATGDGCRCAATGTT	Api_tufA-R2	CCYCTTATYTTTTCYTCWGGAGCWGAATCDATATCAGAA
C	Api_tufA-5F1	GGTACTATWGGWCAYGTWGAYCATGGWAA	Api_tufA-5F2	CATGGWAARACAACWTTAACAACAGCAATATCTTA
	Api_tufA-3R1	GTCATTATAGCTCAATGGTAGAGCMATGGATTGA	Api_tufA-3R2	GAAGATCCATRTGTTATCAGTTCAAATCTGAT
D	Api_tufA-F1	CTAAAGAAGAAGGAGGTCGTCATAAACCTTTTA	Api_tufA-F2	CCTCAATTTTTTATTCATACAGTAGATGTAACTGG
	Api_clpc-R1	GCAAATTCAGTTTTACCAGTACCACTAGGACCA	Api_clpc-R2	CTAGGACCACATAAAATCCAACTACCAATAGGTT
E	Pkn_clpC-5F	CCTATATATTTACATCATACAGAAATATGGGTATTA		
	Pkn_clpC-3R	GGACGAGCTCCATATAAAGGATTATAAGTTAA		
F	Api_clpc-F1	GAYGAAGGTAGAYTAACWGATWCWACRGGTAAATTAA	Api_clpc-F2	CAAGTAAYTTAGGTTGYCCTAAAAATTATRATWYRTAT
	Api_rpoB-F1	CCTATTAAAGGARATACAAARCAAGGWGGTCAAAGATTT	Api_rpoB-F2	GAAATGGARGTATGGGCATTAGAAGCTTTTGGRGCTT
G	Api_rpoB-F3	TATATAAACCTATAGTDTGGGTMGGWGAAA	Api_rpoB-F4	TAGGTAAAATATTAGCTATAAATTCAAATTTATTA
	Api_rpoB-R3	ATAGTATTAGGTAYTAAAACTTCTCCWATWATAA	Api_rpoB-R4	GMAGACCATTTWATTATTTGTTTAGGATTTAAAA
H	Api_sufB-F1	GCAGATACTGGTAGTAAAATGTATCATATAGGATCTT	Api_sufB-F2	GTATCATATAGGATCTTATACTAAAAGTTATATAATTTCA
	Api_rpoB-R1	CCGCATCTTCATATTCATAACCTAAATAAGAACCATA	Api_rpoB-R2	CTAAATAAGAACCATATCCTACTAATAAATTATTTCCTAA
I	Api_lsu-F1	GTCTGTTCGCCTATTAAAGCGATACGTGAGCT	Api_lsu-F2	CGTCGTGAGACAGTTCGGTCCATATCTAATATAAGT
	Api_sufB-R1	GACATWGMTTCTGAMATATTTAATCCACGTTGCAT	Api_sufB-R2	YATAAGGAATWGTHACHGTYAAAGAWTTATYYCCAAAT
J	Pkn_lsu-5F	GAGCATAAGGAAAGTTCGTCGGATTCATGCT		
	Pkn_lsu-3R	CAAGCTAATGATGAGATTTGGACTCATAATCTACTGA		
K	Api_lsu-R1	CAATTCGYTCRCCACTACTATGAAAATCGTTATTACT	Api_lsu-R2	CCTTTAAGTACTAAGATGATTCAATTCCTTAAGTT
	Api_ssu-R1	CGCTTATTGTAAAATATTCCTCACTGCTGATTT	Api_ssu-R2	GGCTTTATTTCAATCCTAATGTGATTGTACATTCT
L	Pkn_ssu-5F	GCGAGTTTGATCCTAGCTTAGAATTAACGCTAGA		
	Api_ssu-R	AGAATGTACAATCACATTAGGATTGAAATAAAGCC		
M	Api_ssu-3F	GATGGAATCACTAGTAATCGCTAATTAGAA		
	Api_Ile-R	ACCCTTATCAAGAGTATGTTTTACCATTAAACTA		

Supplementary Fig. S3 PCR primer sequences and amplified *Plasmodium* apicoplast genome regions.

Table S1 *Plasmodium* species used in this study.

species	strain	ATCC No.	Accession No.[a]	host
Plasmodium cynomolgi	ceylonensis		**AP018101**	Asian macaque
P. cynomolgi	Berok		**AP018102**	Asian macaque
P. knowlesi	H	30158	**AP018103**	Asian macaque
P. fragile	Hackeri		**AP018104**	Asian macaque
P. fieldi	A.b.introlatus	30164	**AP018105**	Asian macaque
P. simiovale		30104	**AP018106**	Asian macaque
P. hylobati	WAK	30154	**AP018107**	Gibbon
P. inui	Celebes		**AP018108**	Asian macaque
P. gonderi		30045	**AP018109**	African guenon
P. ovale	Nigeria II		AB649417	Human
P. malariae	Kisii 67		AB649418	Human
P. vivax	Salvador I		AB649419	Human
P. coatneyi	CDC		AB649420	Asian macaque
P. berghei	ANKA		AB649421	Rodent
P. yoelii	17XNL		AB649422	Rodent
P. chabaudi	AS		AB649423	Rodent
P. falciparum	C10		X95275, X95276	Human
P. gallinaceum	A8		AB649424	Avian

[a] Newly determined in this study were shown in bold.

Table S2 Gene component and gene length of apicoplast genome in nine Plasmodium species that were newly determined in this study.

species	*P. cynomolgi*			*P. cynomolgi*			*P. knowlesi*			*P. fragile*			*P. fieldi*			*P. simiovale*			*P. hylobati*			*P. inui*			*P. gonderi*		
strain	(ceylonensis)			(Berok)			(H, ATCC30158)			(Hackeri)			(ABI. ATCC30164)			(ATCC30104)			(WAK, ATCC30154)			(Celeves)			(ATCC30045)		
Accession No.	AP018101			AP018102			AP018103			AP018104			AP018105			AP018106			AP018107			AP018108			AP018109		
gene[a]	start	end	length	start	end	length	start	end	length	start	end	length	start	end	length	start	end	length	start	end	length	start	end	length	start	end	length
T(ugu)	1	73	73	1	73	73	1	73	73	1	73	73	1	73	73	1	73	73	1	73	73	1	73	73	1	73	73
rps4	100	714	615	100	714	615	100	714	615	100	714	615	100	714	615	100	714	615	100	714	615	100	714	615	100	714	615
H(gug)	726	797	72	726	797	72	726	797	72	726	797	72	727	798	72	726	797	72	726	797	72	727	798	72	730	801	72
C(gca)	820	891	72	820	891	72	818	888	71	821	892	72	820	891	72	822	893	72	820	891	72	822	893	72	828	900	73
L*(uaa)	904	1132	229	904	1132	229	903	1131	229	906	1134	229	905	1133	229	907	1136	230	905	1123	219	907	1126	220	914	1145	232
==> intron	940	1082	143	940	1082	143	939	1081	143	942	1084	143	941	1083	143	943	1086	144	941	1073	133	943	1076	134	950	1095	146
M(cau)	1141	1227	87	1141	1227	87	1140	1226	87	1143	1229	87	1142	1228	87	1145	1231	87	1132	1218	87	1135	1221	87	1154	1240	87
Y(gua)	1240	1323	84	1240	1323	84	1239	1322	84	1242	1325	84	1241	1324	84	1244	1327	84	1230	1313	84	1235	1318	84	1255	1338	84
S(gcu)	1331	1418	88	1331	1418	88	1330	1422	93	1333	1421	89	1334	1422	89	1333	1421	89	1321	1410	90	1326	1415	90	1344	1440	97
D(guc)	1424	1498	75	1424	1498	75	1428	1502	75	1427	1501	75	1428	1502	75	1427	1501	75	1416	1490	75	1421	1495	75	1454	1528	75
K(uuu)	1508	1580	73	1508	1580	73	1512	1584	73	1511	1583	73	1512	1584	73	1511	1583	73	1500	1572	73	1505	1577	73	1538	1610	73
E(uuc)	1594	1663	70	1594	1663	70	1599	1668	70	1597	1666	70	1598	1667	70	1597	1666	70	1586	1655	70	1592	1661	70	1622	1691	70
P(ugg)	1677	1748	72	1677	1748	72	1682	1753	72	1680	1751	72	1681	1752	72	1680	1751	72	1669	1740	72	1675	1746	72	1699	1769	71
rpl4	1777	2382	606	1777	2382	606	1782	2390	609	1780	2385	606	1781	2383	603	1780	2358	579	1769	2377	609	1775	2371	597	1798	2409	612
rpl23	2394	2618	225	2395	2619	225	2398	2622	225	2394	2618	225	2399	2623	225	2366	2590	225	2388	2615	228	2382	2609	228	2420	2638	219
rpl2	2615	3352	738	2616	3353	738	2619	3356	738	2615	3352	738	2620	3357	738	2587	3324	738	2612	3349	738	2606	3343	738	2635	3372	738
rps19	3378	3647	270	3380	3649	270	3371	3640	270	3379	3648	270	3380	3649	270	3351	3620	270	3376	3645	270	3370	3639	270	3407	3670	264
rps3	3651	4295	645	3653	4297	645	3644	4288	645	3652	4296	645	3654	4298	645	3624	4271	648	3649	4293	645	3643	4287	645	3681	4328	648
rpl16	4313	4705	393	4315	4704	390	4306	4698	393	4309	4701	393	4316	4708	393	4283	4675	393	4313	4708	396	4307	4702	396	4340	4735	396
rps17	4722	4946	225	4721	4945	225	4708	4932	225	4714	4938	225	4721	4945	225	4688	4912	225	4721	4945	225	4715	4939	225	4755	4979	225
rpl14	4943	5305	363	4942	5304	363	4929	5291	363	4935	5297	363	4942	5298	357	4909	5271	363	4942	5304	363	4936	5298	363	4976	5338	363
rps8	5308	5694	387	5307	5693	387	5294	5680	387	5302	5688	387	5301	5687	387	5279	5665	387	5307	5693	387	5301	5687	387	5338	5724	387
rpl6	5713	6234	522	5712	6232	521	5702	6214	513	5707	6225	519	5709	6230	522	5685	6206	522	5707	6228	522	5706	6218	513	5737	6255	519
rps5	6231	6953	723	6229	6951	723	6211	6933	723	6222	6947	726	6227	6949	723	6203	6925	723	6225	6926	702	6215	6943	729	6252	6977	726
ORF91	6957	7199	243	6955	7197	243	6940	7182	243	6953	7195	243	6953	7195	243	6929	7171	243	6930	7172	243	6947	7189	243	6988	7230	243
rpl36	7196	7306	111	7194	7304	111	7179	7289	111	7192	7302	111	7192	7302	111	7168	7278	111	7169	7279	111	7186	7296	111	7227	7337	111
rps11	7303	7704	402	7301	7702	402	7286	7987	702	7299	7700	402	7299	7700	402	7275	7676	402	7276	7677	402	7293	7694	402	7334	7735	402
rps12	7698	8075	378	7696	8072	377	7681	8058	378	7694	8071	378	7694	8071	378	7670	8050	381	7671	8048	378	7688	8062	375	7729	8103	375
rps7	8097	8531	435	8094	8528	435	8072	8494	423	8093	8527	435	8094	8519	426	8078	8497	420	8070	8498	429	8088	8522	435	8110	8532	423
tufA	8605	9834	1230	8602	9831	1230	8568	9797	1230	8599	9828	1230	8600	9829	1230	8570	9799	1230	8570	9799	1230	8585	9814	1230	8595	9824	1230
ORF78	9841	10092	252	9838	10089	252	9804	10055	252	9835	10086	252	9836	10084	249	9806	10057	252	9806	10057	252	9821	10075	255	9836	10084	249
F(gaa)	10069	10140	72	10066	10137	72	10032	10103	72	10063	10134	72	10061	10132	72	10034	10105	72	10034	10105	72	10052	10123	72	10061	10132	72
Q(uug)	10160	10231	72	10157	10228	72	10123	10194	72	10154	10225	72	10153	10224	72	10125	10196	72	10125	10196	72	10143	10214	72	10154	10225	72
G(acc)	10242	10311	70	10239	10308	70	10205	10274	70	10236	10305	70	10235	10304	70	10207	10276	70	10207	10276	70	10225	10294	70	10233	10302	70
W(cca)	10327	10397	71	10324	10394	71	10288	10358	71	10319	10389	71	10320	10390	71	10292	10362	71	10292	10362	71	10310	10380	71	10317	10387	71
ORF129	10433	10810	378	10430	10807	378	10394	10771	378	10425	10802	378	10422	10799	378	10398	10784	387	10394	10771	378	10418	10816	399	10425	10802	378
clpC	10817	13108	2292	10814	13108	2295	10778	13066	2289	10809	13097	2289	10806	13097	2292	10781	13063	2283	10778	13063	2286	10803	13088	2286	10810	13098	2289
G(ucc)	13112	13184	73	13112	13184	73	13070	13142	73	13101	13173	73	13101	13173	73	13067	13139	73	13067	13139	73	13092	13164	73	13102	13174	73
ORF79	13216	13446	231	13216	13446	231	13173	13403	231	13206	13436	231	13203	13433	231	13169	13402	234	13169	13399	231	13194	13424	231	13200	13466	267
S(uga)	13455	13542	88	13455	13542	88	13413	13500	88	13444	13529	86	13441	13528	88	13410	13497	88	13407	13494	88	13432	13519	88	13435	13521	87
ORF105	13570	13881	312	13570	13881	312	13526	13837	312	13557	13868	312	13556	13867	312	13525	13836	312	13522	13833	312	13548	13859	312	13553	13864	312
rps2	13868	14548	681	13868	14548	681	13824	14504	681	13855	14535	681	13854	14534	681	13823	14503	681	13820	14500	681	13846	14526	681	13851	14531	681
rpoD	14562	17482	2921	14562	17479	2918	14518	17456	2939	14548	17471	2924	14547	17472	2926	14516	17436	2921	14514	17452	2939	14541	17488	2948	14544	17442	2899
rpoC	17492	19213	1722	17489	19210	1722	17466	19187	1722	17481	19202	1722	17482	19203	1722	17446	19167	1722	17462	19183	1722	17498	19219	1722	17452	19170	1719
rpoB	19224	22289	3066	19221	22286	3066	19191	22256	3066	19206	22271	3066	19207	22275	3069	19171	22239	3069	19187	22255	3069	19223	22288	3066	19174	22239	3066
ORF101	22297	22590	294	22294	22587	294	22264	22557	294	22280	22573	294	22276	22569	294	22236	22535	300	22263	22556	294	22296	22589	294	22249	22560	312
ORF51	22612	22788	177	22609	22785	177	22579	22749	171	22595	22771	177	22593	22769	177	22569	22748	180	22579	22749	171	22615	22791	177	22588	22764	177
sufB	22785	24194	1410	22782	24191	1410	22746	24155	1410	22768	24177	1410	22766	24175	1410	22745	24154	1410	22746	24155	1410	22788	24197	1410	22761	24170	1410
T(ugu)	24232	24304	73	24229	24301	73	24194	24266	73	24215	24287	73	24213	24285	73	24192	24264	73	24193	24265	73	24235	24307	73	24197	24269	73
LSUrRNA	24318	27013	2696	24315	27010	2696	24280	26975	2696	24301	26995	2695	24299	26999	2701	24278	26971	2694	24279	26979	2701	24321	27025	2705	24283	26983	2701
M(cau)	27059	27132	74	27056	27129	74	27021	27094	74	27040	27113	74	27045	27118	74	27018	27091	74	27025	27098	74	27072	27145	74	27035	27108	74
R'(acg)	27142	27213	72	27139	27210	72	27104	27175	72	27123	27194	72	27128	27199	72	27101	27172	72	27108	27179	72	27155	27226	72	27118	27189	72
V(uac)	27236	27307	72	27233	27304	72	27198	27269	72	27217	27288	72	27222	27293	72	27195	27266	72	27202	27273	72	27249	27320	72	27212	27283	72
R(ucu)	27311	27382	72	27308	27379	72	27273	27344	72	27292	27363	72	27297	27368	72	27270	27341	72	27277	27348	72	27324	27395	72	27287	27358	72
L(uag)	27389	27466	78	27386	27463	78	27355	27432	78	27374	27451	78	27379	27456	78	27352	27429	78	27362	27439	78	27406	27483	78	27369	27445	77
N(guu)	27485	27556	72	27482	27553	72	27451	27522	72	27470	27541	72	27475	27546	72	27448	27519	72	27458	27529	72	27502	27573	72	27466	27537	72
A(ugc)	27586	27656	71	27583	27653	71	27555	27625	71	27571	27641	71	27577	27647	71	27549	27619	71	27561	27631	71	27605	27675	71	27567	27637	71
SSUrRNA	27699	29158	1460	27696	29155	1460	27668	29124	1457	27686	29143	1458	27694	29144	1451	27662	29119	1458	27675	29127	1453	27719	29173	1455	27680	29132	1453
Ile(gau)	29315	29341	27	29312	29382	71	29273	29299	27	29302	29331	30	29307	29331	25	29280	29309	30	29280	29303	24	29331	29354	24	-	-	
A+T %		86.92			86.92			86.53			86.93			86.78			86.90			86.89			87.11			87.02	

a Genes incomplemental chain are shown in blue, and genes in the inverted repeat regions are shaded in gray.

Table S3 Sequence similarities of *Plasmodium* apicoplast genes.

gene[b]	similarity[a] aa (%) 18 OTUs	similarity[a] nuc(%) 18 OTUs	gene[b]	similarity[a] aa (%) 18 OTUs	similarity[a] nuc(%) 18 OTUs
rps4	84.9	90.4	ORF78	83.1	91.3
tRNA-H(gug)	-	96.8	tRNA-F(gaa)	-	98.1
tRNA-C(gca)	-	95.3	tRNA-Q(uug)	-	98.8
tRNA-L(uaa)[c]	-	97.1	tRNA-G(acc)	-	96.5
tRNA-M(cau)	-	95.3	tRNA-W(cca)	-	96.0
tRNA-Y(gua)	-	96.6	ORF129	81.9	89.6
tRNA-S(gcu)	-	94.5	clpC	84.0	89.5
tRNA-D(guc)	-	97.1	tRNA-G(ucc)	-	96.5
tRNA-K(uuu)	-	97.3	ORF79	82.5	89.6
tRNA-E(uuc)	-	98.7	tRNA-S(uga)	-	96.0
tRNA-P(ugg)	-	97.7	ORF105	78.2	88.1
rpl4	75.1	87.2	rps2	84.4	90.9
rpl23	71.0	84.5	rpoD	81.4	88.1
rpl2	89.1	91.5	rpoC	90.4	92.6
rps19	87.4	92.3	rpoB	86.2	90.6
rps3	81.6	89.4	ORF101	76.2	87.1
rpl16	78.7	86.0	ORF51	76.4	86.3
rps17	73.9	84.9	sufB	90.5	92.5
rpl14	78.7	86.0	tRNA-T(ugu)	-	96.0
rps8	80.5	88.8	LSUrRNA	-	95.8
rpl6	74.4	85.5	tRNA-M(cau)	-	100.0
rps5	75.3	85.6	tRNA-R(acg)	-	93.5
ORF91	77.0	86.9	tRNA-V(uac)	-	98.6
rpl36	94.9	95.8	tRNA-R(ucu)	-	99.8
rps11	86.9	90.8	tRNA-L(uag)	-	96.7
rps12	90.8	90.8	tRNA-N(guu)	-	97.7
rps7	87.3	92.4	tRNA-A(ugc)	-	98.3
tufA	91.2	91.5	SSUrRNA	-	96.0

[a] Avarage value of pairwise comparison of18 OTUs.
[b] Protein coding genes are shown in blue.
[c] Intron was removed from the comparison because of the ambiguos alignment.

Table S4 Gene ID of *Plasmodium vivax* . These genes and their orthologs of six *Plasmodium* species, *P. coatney, P. knowlesi, P. fragile, P. inui, P. cynomolgi* and *P. gonderi* were used for the phylogenetic analysis.

ID_chr1	gene	No of aa position used for the phylogeny	ID_chr2	gene	No of aa position used for the phylogeny	ID_chr3	gene	No of aa position used for the phylogeny	ID_chr4	gene	No of aa position used for the phylogeny
**PVX_087670**	hypothetical protein, conserved	113	**PVX_081200**	hypothetical protein, conserved	75	**PVX_000005**	Plasmodium exported protein, unknown function	224	**PVX_002550**	hypothetical protein, conserved	154
**PVX_087680**	chitinase(CHT1)	567	**PVX_081205**	TatD-like deoxyribonuclease, putative	396	**PVX_000525**	protein kinase domain containing protein	1684	**PVX_002555**	Plasmodium exported protein, unknown function	239
**PVX_087685**	centrin	106	**PVX_081210**	hypothetical protein, conserved	79	**PVX_000530**	hypothetical protein, conserved	4307	**PVX_002575**	replication factor C subunit 1, putative	731
**PVX_087690**	hypothetical protein, conserved	1205	**PVX_081215**	hypothetical protein, conserved	297	**PVX_000535**	thioredoxin, putative	422	**PVX_002580**	pseudouridine synthetase, putative	278
**PVX_087695**	hypothetical protein, conserved	177	**PVX_081220**	hypothetical protein, conserved	115	**PVX_000540**	hypothetical protein, conserved	757	**PVX_002585**	ribosome associated membrane protein RAMP4, putative	72
**PVX_087700**	hypothetical protein, conserved	1044	**PVX_081225**	hypothetical protein, conserved	213	**PVX_000545**	serine--tRNA ligase, putative	454	**PVX_002590**	40S ribosomal protein S30, putative	57
**PVX_087705**	hypothetical protein, conserved	129	**PVX_081230**	UMP-CMP kinase, putative	222	**PVX_000550**	hypothetical protein, conserved	651	**PVX_002595**	hypothetical protein, conserved	126
**PVX_087710**	hypothetical protein, conserved	606	**PVX_081235**	hypothetical protein, conserved	438	**PVX_000555**	calcium-dependent protein kinase 4, putative (CDPK4)	520	**PVX_002600**	hypothetical protein, conserved	421
**PVX_087715**	erythrocyte membrane-associated antigen	1813	**PVX_081240**	replication factor c, putative	789	**PVX_000560**	queuine tRNA-ribosyltransferase, putative	494	**PVX_002605**	nuclear protein SkiP, putative	444
**PVX_087720**	hypothetical protein, conserved	261	**PVX_081245**	hypothetical protein, conserved	83	**PVX_000565**	hypothetical protein, conserved	328	**PVX_002610**	hypothetical protein, conserved	1082
**PVX_087725**	hypothetical protein, conserved	170	**PVX_081250**	kinesin-8, putative	950	**PVX_000570**	hypothetical protein, conserved	226	**PVX_002615**	small nuclear ribonucleoprotein Sm D2, putative	99
**PVX_087730**	hypothetical protein, conserved	549	**PVX_081255**	adenylate kinase-like protein 1, putative (AKLP1)	173	**PVX_000575**	hypothetical protein, conserved	616	**PVX_002620**	DEAD/DEAH box helicase, putative	458
**PVX_087735**	hypothetical protein, conserved	2803	**PVX_081260**	transcription initiation factor TFIIB, putative	361	**PVX_000580**	hypothetical protein, conserved	169	**PVX_002625**	rRNA methylase, putative	126
**PVX_087740**	hypothetical protein, conserved	2248	**PVX_081265**	chromatin assembly factor 1 protein WD40 domain, putative	446	**PVX_000585**	transporter/permease protein, putative	321	**PVX_002630**	hypothetical protein	280
**PVX_087745**	hypothetical protein, conserved	1751	**PVX_081270**	phosphatidylinositol-4-phosphate 5-kinase, putative (PIP5K)	870	**PVX_000590**	eukaryotic translation initiation factor 3 subunit 2, putative	326	**PVX_002635**	hypothetical protein	242
**PVX_087750**	tRNA m5C-methyltransferase	853	**PVX_081275**	bromodomain protein, putative	409	**PVX_000595**	hypothetical protein, conserved	541	**PVX_002640**	replication factor C subunit 2, putative	329
**PVX_087755**	hypothetical protein, conserved	1147	**PVX_081277**	DNA-directed RNA polymerase 2, putative	161	**PVX_000600**	cysteine desulfurase, putative (SufS)	482	**PVX_002645**	hypothetical protein, conserved	450
**PVX_087760**	phosphoinositide-binding protein	612	**PVX_081280**	FAD-linked sulfhydryl oxidase ERV1, putative (ERV1)	136	**PVX_000604**	hypothetical protein, conserved	427	**PVX_002650**	40S ribosomal protein S26, putative (RPS26)	106
**PVX_087765**	serine-threonine protein kinase	723	**PVX_081285**	selenocysteine-specific elongation factor selB homologue, putative	487	**PVX_000606**	transcription initiation factor IIA subunit 1, putative	147	**PVX_002655**	hypothetical protein, conserved	203
**PVX_087775**	ubiquitin transferase	2451	**PVX_081290**	HP	149	**PVX_000610**	hypothetical protein, conserved	367	**PVX_002660**	hypothetical protein, conserved	1110
**PVX_087780**	hypothetical protein, conserved	879	**PVX_081295**	hypothetical integral membrane protein, DUF56 family, putative	677	**PVX_000615**	hypothetical protein, conserved	318	**PVX_002665**	calcium-dependent protein kinase 1, putative (CDPK1)	513
**PVX_087785**	hypothetical protein, conserved	850	**PVX_081300**	phenylalanyl-tRNA synthetase beta chain, putative	515	**PVX_000620**	protein SDA1, putative (SDA1)	722	**PVX_002670**	hypothetical protein, conserved	144
**PVX_087790**	peptide chain release factor	333	**PVX_081305**	HP	182	**PVX_000625**	RNA-binding protein, putative	580	**PVX_002675**	AP-2 complex subunit sigma, putative	140
**PVX_087795**	hypothetical protein, conserved	838	**PVX_081307**	cold-shock protein, putative	59	**PVX_000630**	phosphopantothenate--cysteine ligase, putative,phosphopantothenoylcystei ne synthetase, putative	290	**PVX_002680**	hypothetical protein, conserved	803
**PVX_087800**	hypothetical protein, conserved	366	**PVX_081310**	N-acetyltransferase, putative	158	**PVX_000635**	hypothetical protein, conserved	282	**PVX_002685**	ATP synthase alpha chain, putative	517
**PVX_087802**	hypothetical protein, conserved	956	**PVX_081315**	tubulin-specific chaperone a, putative	144	**PVX_000640**	30S ribosomal protein S12, putative	203	**PVX_002690**	hypothetical protein, conserved	683
**PVX_087805**	origin recognition complex subunit 2	580	**PVX_081320**	elongation of very long chain fatty acids protein 3, putative	308	**PVX_000645**	hypothetical protein, conserved	513	**PVX_002695**	hypothetical protein, conserved	290
**PVX_087810**	minchromosome maintenance (MCM) complex subunit	806	**PVX_081325**	mRNA cleavage factor-like protein, putative	209	**PVX_000650**	DNA polymerase alpha, putative	1348	**PVX_002700**	hypothetical protein, conserved	800
**PVX_087815**	inositol-phosphate phosphatase	1563	**PVX_081330**	LCCL domain-containing protein (CCp5)	914	**PVX_000660**	hypothetical protein	2442	**PVX_002705**	hypothetical protein, conserved	1031
**PVX_087820**	RNA helicase	755	**PVX_081335**	HP	153	**PVX_000670**	hypothetical protein, conserved	389	**PVX_002710**	hypothetical protein, conserved	308
**PVX_087825**	40S ribosomal protein S29	53	**PVX_081340**	hypothetical protein, conserved	337	**PVX_000675**	hypothetical protein	113	**PVX_002715**	hypothetical protein, conserved	658
**PVX_087830**	cysteine-rich secretory protein	157	**PVX_081345**	secreted ookinete protein, putative (PSOP24)	338	**PVX_000680**	RNA helicase, putative	762	**PVX_002720**	vacuolar protein sorting-associated protein 45, putative	622

ID_chr1	gene	No of aa position used for the phylogeny	ID_chr2	gene	No of aa position used for the phylogeny	ID_chr3	gene	No of aa position used for the phylogeny	ID_chr4	gene	No of aa position used for the phylogeny
**PVX_087835**	ATP synthase subunit C	163	**PVX_081350**	hypothetical protein, conserved	190	**PVX_000685**	hypothetical protein, conserved	309	**PVX_002725**	hypothetical protein, conserved	706
**PVX_087840**	hypothetical protein, conserved	1118	**PVX_081355**	hypothetical protein, conserved	49	**PVX_000690**	hypothetical protein, conserved	646	**PVX_002730**	hypothetical protein, conserved	184
**PVX_087845**	hypothetical protein, conserved	1199	**PVX_081360**	hypothetical protein, conserved	212	**PVX_000695**	hypothetical protein, conserved	193	**PVX_002735**	hypothetical protein	552
**PVX_087851**	hypothetical protein, conserved	48	**PVX_081365**	hypothetical protein, conserved	903	**PVX_000700**	hypothetical protein, conserved	232	**PVX_002740**	DEAD/DEAH helicase, putative	1086
**PVX_087855**	hypothetical protein, conserved	38	**PVX_081370**	hypothetical protein, conserved	425	**PVX_000705**	hypothetical protein, conserved	634	**PVX_002745**	palmitoyltransferase, putative (DHHC11)	255
**PVX_087860**	60S ribosomal protein L37	91	**PVX_081375**	proteasome subunit beta type-3, putative	187	**PVX_000710**	hypothetical protein, conserved	812	**PVX_002750**	origin recognition complex subunit 5, putative (ORC5)	648
**PVX_087865**	hypothetical protein, conserved	725	**PVX_081380**	hypothetical protein, conserved	168	**PVX_000715**	ribosome biogenesis GTPase A, putative (RbgA)	576	**PVX_002755**	DNA-directed RNA polymerase II 135 kDa polypeptide, putative	1293
**PVX_087870**	hypothetical protein, conserved	501	**PVX_081385**	double-strand break repair protein MRE11, putative	572	**PVX_000720**	hypothetical protein, conserved	312	**PVX_002760**	hypothetical protein, conserved	189
**PVX_087875**	DNA mismatch repair protein, putative (MSH2-2)	764	**PVX_081390**	hypothetical protein, conserved	335	**PVX_000725**	hypothetical protein, conserved	197	**PVX_002765**	hypothetical protein, conserved	441
**PVX_087880**	hypothetical protein, conserved	1087	**PVX_081400**	patched family protein, putative	1131	**PVX_000730**	exosome complex component RRP4, putative (RRP4)	327	**PVX_002770**	hypothetical protein, conserved	455
**PVX_087885**	rhoptry associated membrane antigen	468	**PVX_081405**	hypothetical protein, conserved	159	**PVX_000735**	protein phosphatase 2C, putative	653	**PVX_002775**	hypothetical protein, conserved	214
**PVX_087895**	AAA family ATPase	545	**PVX_081410**	carbon catabolite repressor protein 4, putative	316	**PVX_000750**	actin-like protein, putative	403	**PVX_002785**	ATP-dependent acyl-CoA synthetase, putative	695
**PVX_087900**	hypothetical protein, conserved	972	**PVX_081415**	hypothetical protein, conserved	327	**PVX_000755**	zinc finger protein, putative	423	**PVX_002790**	rhoptry neck protein 6, putative (RON6)	383
**PVX_087905**	hypothetical protein, conserved	209	**PVX_081420**	centrin-1, putative (CEN1)	167	**PVX_000760**	hypothetical protein, conserved	181	**PVX_002795**	hypothetical protein, conserved	972
**PVX_087910**	ubiquitin-protein ligase e3	552	**PVX_081425**	hypothetical protein, conserved	383	**PVX_000765**	replication factor a protein, putative	838	**PVX_002800**	hypothetical protein, conserved	293
**PVX_087915**	RAP protein	682	**PVX_081430**	ras-related protein Rab-5C, putative (RAB5c)	212	**PVX_000770**	hypothetical protein, conserved	341	**PVX_002805**	calcium-dependent protein kinase 4, putative	639
**PVX_087920**	TGF beta-inducible nuclear protein	258	**PVX_081435**	ribosome biogenesis protein BMS1, putative	939	**PVX_000775**	chaperone DNAJ protein, putative	480	**PVX_002810**	hypothetical protein, conserved	252
**PVX_087925**	cytoskeleton associated protein	570	**PVX_081445**	hypothetical protein, conserved	333	**PVX_000780**	hypothetical protein, conserved	708	**PVX_002815**	hypothetical protein, conserved	365
**PVX_087930**	hypothetical protein, conserved	103	**PVX_081450**	pre-rRNA-processing protein TSR2, putative	154	**PVX_000785**	ribosomal processing protein, putative	455	**PVX_002820**	hypothetical protein	477
**PVX_087935**	DNA-directed RNA polymerase II	68	**PVX_081455**	calcium-transporting ATPase, putative	1050	**PVX_000790**	U4/U6 small nuclear ribonucleoprotein PRP31, putative	478	**PVX_002825**	50S ribosomal protein L13, putative	211
**PVX_087940**	hypothetical protein, conserved	645	**PVX_081460**	hypothetical protein, conserved	192	**PVX_000795**	hypothetical protein, conserved	1620	**PVX_002830**	protein transport protein SEC31, putative (SEC31)	1160
**PVX_087945**	O-sialoglycoprotein endopeptidase	232	**PVX_081465**	vacuolar ATP synthase subunit c, putative	382	**PVX_000800**	tRNA N6-adenosine threonylcarbamoyltransferase, putative (KAE1)	656	**PVX_002835**	T-complex protein 1, theta subunit, putative	542
**PVX_087950**	HSP86	695	**PVX_081470**	hypothetical protein, conserved	593	**PVX_000805**	hypothetical protein, conserved	250	**PVX_002840**	hypothetical protein, conserved	482
**PVX_087955**	HSP86 family	343	**PVX_081475**	DNA binding protein, putative	286	**PVX_000810**	perforin-like protein 1 (PLP1)	739	**PVX_002850**	hypothetical protein, conserved	153
**PVX_087960**	inner membrane complex protein 1d	205	**PVX_081480**	hypothetical protein, conserved	684	**PVX_000815**	sporozoite invasion-associated protein 1, putative (SIAP1)	943	**PVX_002855**	hypothetical protein, conserved	1476
**PVX_087965**	Cg8 protein	204	**PVX_081485**	hypothetical protein	608	**PVX_000820**	flap endonuclease 1, putative (FEN1)	531	**PVX_002860**	hypothetical protein, conserved	381
**PVX_087970**	HSP70-z	819	**PVX_081490**	hypothetical protein, conserved	895	**PVX_000825**	hypothetical protein, conserved	373	**PVX_002865**	serine/threonine protein kinase, putative	338
**PVX_087975**	Cg3 protein	205	**PVX_081495**	hypothetical protein, conserved	310	**PVX_000830**	hypothetical protein, conserved	180	**PVX_002867**	conserved Plasmodium protein, unknown function	47
**PVX_087980**	chloroquine resistance transporter	395	**PVX_081498**	hypothetical protein, conserved	117	**PVX_000835**	hypothetical protein, conserved	389	**PVX_002870**	hypothetical protein, conserved	121
**PVX_087985**	Cg1 protein	673	**PVX_081502**	hypothetical protein, conserved	161	**PVX_000840**	hypothetical protein, conserved	689	**PVX_002875**	heat shock protein, putative	324
**PVX_087990**	glutaredoxin-like protein (GLP3)	161	**PVX_081505**	hypothetical protein, conserved	518	**PVX_000845**	hypothetical protein, conserved	142	**PVX_002880**	hypothetical protein, conserved	165
**PVX_087995**	Cg2 protein	1487	**PVX_081510**	hypothetical protein, conserved	136	**PVX_000850**	AAA family ATPase, putative	569	**PVX_002885**	Leu/Phe-tRNA protein transferase, putative	289
**PVX_088000**	Cg7 protein	721	**PVX_081515**	transporter, putative	443	**PVX_000855**	hypothetical protein, conserved	766	**PVX_002890**	hypothetical protein, conserved	446
**PVX_088005**	hypothetical protein, conserved	129	**PVX_081520**	transporter, putative	475	**PVX_000860**	hypothetical protein, conserved	970	**PVX_002895**	hypothetical protein, conserved	253
**PVX_088007**	ribonucleases p-mrp protein subunit	610	**PVX_081525**	hypothetical protein, conserved	749	**PVX_000865**	hypothetical protein, conserved	455	**PVX_002900**	secreted protein with altered thrombospondin repeat domain, putative (SPATR)	251
**PVX_088010**	lysophospholipase	318	**PVX_081530**	hypothetical protein, conserved	201	**PVX_000870**	mitochondrial carrier protein, putative	379	**PVX_002905**	hypothetical protein, conserved	2405
**PVX_088015**	hypothetical protein, conserved	325	**PVX_081535**	4-hydroxy-3-methylbut-2-enyl diphosphate reductase, putative	313	**PVX_000875**	hypothetical protein, conserved	107	**PVX_002910**	hypothetical protein, conserved	2517

ID_chr1	gene	No of aa position used for the phylogeny	ID_chr2	gene	No of aa position used for the phylogeny	ID_chr3	gene	No of aa position used for the phylogeny	ID_chr4	gene	No of aa position used for the phylogeny
**PVX_088020**	hypothetical protein, conserved	64	**PVX_081540**	ubiquitin carboxyl-terminal hydrolase 1, putative (UBP1)	1426	**PVX_000880**	hypothetical protein, conserved	229	**PVX_002915**	eukaryotic peptide chain release factor subunit 1, putative	402
**PVX_088025**	hypothetical protein, conserved	1240	**PVX_081550**	StAR-related lipid transfer protein, putative	314	**PVX_000885**	peptidyl-tRNA hydrolase, putative	190	**PVX_002920**	50S ribosomal protein L7/L12, putative	187
**PVX_088035**	hypothetical protein, conserved	2514	**PVX_081555**	hypothetical protein, conserved	742	**PVX_000890**	methyltransferase, putative	297	**PVX_002925**	hypothetical protein, conserved	400
**PVX_088040**	hypothetical protein, conserved	387	**PVX_081560**	hypothetical protein, conserved	156	**PVX_000895**	hypothetical protein, conserved	197	**PVX_002930**	GDP-fucose transporter, putative	298
**PVX_088045**	hypothetical protein, conserved	659	**PVX_081565**	hypothetical protein, conserved	415	**PVX_000900**	hypothetical protein, conserved	1115	**PVX_002935**	hypothetical protein, conserved	337
**PVX_088050**	hypothetical protein, conserved	203	**PVX_081570**	actin-related protein (ARP1)	378	**PVX_000905**	hypothetical protein, conserved	283	**PVX_002940**	asparagine--tRNA ligase, putative	545
**PVX_088055**	hypothetical protein, conserved	268	**PVX_081572**	L-seryl-tRNA(Sec) kinase, putative (PSTK)	364	**PVX_000910**	hypothetical protein, conserved	822	**PVX_002945**	tyrosine kinase-like protein, putative (TKL1)	732
**PVX_088060**	hypothetical protein, conserved	232	**PVX_081575**	hypothetical protein, conserved	639	**PVX_000915**	hypothetical protein, conserved	138	**PVX_002950**	hypothetical protein, conserved	201
**PVX_088065**	60S ribosomal protein L34a	139	**PVX_081580**	hypothetical protein, conserved	971	**PVX_000920**	hypothetical protein, conserved	568	**PVX_002955**	hypothetical protein, conserved	858
**PVX_088070**	hypothetical protein, conserved	55	**PVX_081585**	hypothetical protein, conserved	599	**PVX_000925**	hydroxyacyl glutathione hydrolase, putative	262	**PVX_002960**	beta-ketoacyl-acyl carrier protein synthase III precursor, putative	371
**PVX_088075**	hypothetical protein, conserved	216	**PVX_081590**	hypothetical protein, conserved	532	**PVX_000930**	sexual stage antigen s16, putative	103	**PVX_002965**	hypothetical protein, conserved	291
**PVX_088080**	mitochondrial ribosomal protein L1 precursor	266	**PVX_081595**	nucleoside transporter 4 (NT4)	431	**PVX_000935**	vacuolar ATP synthase subunit b, putative	493	**PVX_002970**	ras-related protein Rab-5A, putative (RAB5a)	224
**PVX_088085**	cell division cycle ATPase	792	**PVX_081600**	vacuolar protein sorting-associated protein 51, putative (VPS51)	837	**PVX_000940**	hypothetical protein, conserved	369	**PVX_002975**	hypothetical protein, conserved	1061
**PVX_088090**	hypothetical protein, conserved	138	**PVX_081605**	vacuolar protein sorting-associated protein VTA1, putative	212	**PVX_000945**	apical sushi protein, putative (ASP)	539	**PVX_003540**	Plasmodium exported protein (PHISTc), unknown function	125
**PVX_088095**	hypothetical protein, conserved	558	**PVX_081610**	aspartate--tRNA ligase, putative	538	**PVX_000950**	hypothetical protein, conserved	102	**PVX_003545**	Plasmodium exported protein, unknown function	164
**PVX_088100**	hypothetical protein, conserved	96	**PVX_081615**	hypothetical protein, conserved	377	**PVX_000955**	lysine decarboxylase, putative	1395	**PVX_003560**	hypothetical protein	290
**PVX_088105**	sin3 associated polypeptide p18-like protein	117	**PVX_081620**	DNA mismatch repair protein PMS1, putative (PMS1)	748	**PVX_000960**	secy-independent transporter protein, putative	314	**PVX_003570**	hypothetical protein, conserved	663
**PVX_088110**	hypothetical protein, conserved	572	**PVX_081625**	hypothetical protein, conserved	2727	**PVX_000965**	hypothetical protein, conserved	223	**PVX_003575**	octaprenyl pyrophosphate synthase, putative (OPP)	518
**PVX_088115**	coatomer epsilon subunit	272	**PVX_081630**	ubiquitin carboxyl-terminal hydrolase family 2, putative	1732	**PVX_000970**	pre-mRNA-processing-splicing factor 8, putative	2622	**PVX_003578**	conserved Plasmodium protein, unknown function	118
**PVX_088120**	ubiquitin regulatory protein	229	**PVX_081635**	hypothetical protein, conserved	433	**PVX_000975**	liver specific protein 2, putative (LISP2)	634	**PVX_003580**	palmitoyltransferase, putative (DHHC12)	224
**PVX_088125**	plasmepsin X	479	**PVX_081640**	hypothetical protein, conserved	119	**PVX_000980**	Plasmodium yoelii blood stage membrane protein ag-1, putative	430	**PVX_003585**	hypothetical protein, conserved	1507
**PVX_088130**	hypothetical protein, conserved	729	**PVX_081645**	defender against cell death 2, putative	106	**PVX_000985**	protein transport protein Sec24B, putative (SEC24B)	1088	**PVX_003590**	serine/threonine-specific protein kinase, putative	716
**PVX_088140**	hypothetical protein, conserved	620	**PVX_081650**	mitochondrial import inner membrane translocase subunit TIM50, putative (TIM50)	332	**PVX_000990**	ATP-dependent RNA helicase protein, putative	520	**PVX_003595**	hypothetical protein, conserved	280
**PVX_088145**	tyrosyl-tRNA synthetase	349	**PVX_081655**	vacuolar protein sorting-associated protein 53, putative (VPS53)	729	**PVX_000995**	6-cysteine protein (P41)	338	**PVX_003600**	ERCC1 nucleotide excision repair protein, putative	205
**PVX_088150**	proteasome subunit alpha type 5	297	**PVX_081660**	hypothetical protein, conserved	661	**PVX_001000**	hypothetical protein, conserved	282	**PVX_003605**	hypothetical protein, conserved	90
**PVX_088165**	hypothetical protein, conserved	467	**PVX_081665**	cysteine desulfurase, putative	478	**PVX_001005**	dipeptidyl aminopeptidase 3, putative (DPAP3)	527	**PVX_003610**	hypothetical protein, conserved	418
**PVX_088170**	proteasome subunit alpha	259	**PVX_081670**	DNA (cytosine-5)-methyltransferase, putative (DNMT)	543	**PVX_001010**	hypothetical protein, conserved	1523	**PVX_003615**	hypothetical protein, conserved	269
**PVX_088175**	hypothetical protein, conserved	171	**PVX_081675**	proteasome subunit alpha type-5, putative	255	**PVX_001015**	hypothetical protein, conserved	316	**PVX_003620**	hypothetical protein, conserved	543
**PVX_088180**	GTPase, Rab18	200	**PVX_081680**	hypothetical protein, conserved	568	**PVX_001020**	6-cysteine protein (P52)	409	**PVX_003625**	5'-3' exonuclease, N-terminal resolvase-like domain, putative	315
**PVX_088185**	hypothetical protein, conserved	372	**PVX_081685**	hypothetical protein, conserved	782	**PVX_001025**	6-cysteine protein (P36)	338	**PVX_003630**	hypothetical protein, conserved	595
**PVX_088190**	RNA helicase	617	**PVX_081690**	hypothetical protein, conserved	640	**PVX_001030**	hypothetical protein, conserved	350	**PVX_003635**	hypothetical protein, conserved	1347
**PVX_088195**	gas41 homologue	216	**PVX_081695**	cation transporting ATPase, putative	783	**PVX_001035**	hypothetical protein, conserved	152	**PVX_003640**	hypothetical protein, conserved	262
**PVX_088200**	hypothetical protein, conserved	277	**PVX_081700**	hypothetical protein, conserved	1151	**PVX_001040**	transcription factor with AP2 domain(s), putative (ApiAP2)	295	**PVX_003645**	hypothetical protein, conserved	306
**PVX_088205**	vacuolar proton translocating ATPase subunit A	828	**PVX_081705**	eukaryotic translation initiation factor 2 alpha subunit, putative	328	**PVX_001045**	hypothetical protein, conserved	88	**PVX_003650**	dynein light polypeptide 4, axonemal, putative	106
**PVX_088210**	hypothetical protein, conserved	192	**PVX_081707**	conserved Plasmodium protein, unknown function	3423	**PVX_001050**	SET domain protein, putative (SET8)	922	**PVX_003655**	aspartate aminotransferase, mitochondrial precursor, putative	401
**PVX_088215**	hypothetical protein, conserved	1193	**PVX_081710**	actin-like protein, putative (ALP3)	392	**PVX_001055**	alpha/beta hydrolase, putative	402	**PVX_003660**	5'-3' exoribonuclease, putative	662

ID_chr1	gene	No of aa position used for the phylogeny	ID_chr2	gene	No of aa position used for the phylogeny	ID_chr3	gene	No of aa position used for the phylogeny	ID_chr4	gene	No of aa position used for the phylogeny
**PVX_088220**	kinesin-like protein	399	**PVX_081715**	hypothetical protein, conserved	194	**PVX_001060**	splicing factor, putative,CGI-201 protein, short form, putative,crooked neck-like protein 1,	664	**PVX_003665**	hexose transporter	487
**PVX_088225**	DnaJ protein	429	**PVX_081720**	hypothetical protein, conserved	122	**PVX_001065**	hypothetical protein, conserved	586	**PVX_003670**	3'-5' exonuclease domain containing protein	342
**PVX_088230**	glycosyltransferase	145	**PVX_081722**	conserved Plasmodium protein, unknown function	130	**PVX_001070**	hypothetical protein	658	**PVX_003675**	ubiquinone biosynthesis methyltransferase, putative	312
**PVX_088235**	ferlin like protein	1464	**PVX_081725**	hypothetical protein, conserved	726	**PVX_001075**	ubiquitin specific protease, putative	380	**PVX_003685**	hypothetical protein	78
**PVX_088240**	hypothetical protein, conserved	821	**PVX_081730**	alpha/beta hydrolase, putative	441	**PVX_001080**	hypothetical protein, conserved	2451	**PVX_003690**	hypothetical protein, conserved	144
**PVX_088245**	hypothetical protein, conserved	63	**PVX_081740**	hypothetical protein, conserved	352	**PVX_001085**	hypothetical protein, conserved	138	**PVX_003695**	hypothetical protein, conserved	425
**PVX_088250**	AAA family ATPase	703	**PVX_081745**	RNA-binding protein, putative	392	**PVX_001090**	hypothetical protein, conserved	135	**PVX_003700**	hypothetical protein, conserved	442
**PVX_088254**	hypothetical protein, conserved	176	**PVX_081750**	signal recognition particle subunit SRP9, putative (SRP9)	102	**PVX_001095**	hypothetical protein, conserved	471	**PVX_003705**	DNA-directed RNA polymerase II 16 kDa subunit, putative	131
**PVX_088256**	hypothetical protein, conserved	317	**PVX_081755**	hypothetical protein, conserved	1776	**PVX_096015**	Plasmodium exported protein, unknown function	215	**PVX_003715**	hypothetical protein, conserved	559
**PVX_088265**	serine/threonine protein kinase, FIKK family (TSTK0)	713	**PVX_081760**	peroxiredoxin, putative	236	**PVX_096070**	early transcribed membrane protein (ETRAMP)	78	**PVX_003720**	hypothetical protein, conserved	204
**PVX_088270**	apicoplast phosphatidic acid phosphatase	260	**PVX_081765**	60S ribosomal export protein NMD3, putative (NMD3)	674	**PVX_096071**	conserved Plasmodium protein, unknown function	103	**PVX_003725**	PH domain containing protein	118
**PVX_088275**	hypothetical protein, conserved	945	**PVX_081770**	hypothetical protein, conserved	272	**PVX_096075**	hypothetical protein, conserved	598	**PVX_003730**	26S proteasome regulatory subunit RPN1, putative (RPN1)	903
**PVX_088280**	acetyltransferase	209	**PVX_081775**	hypothetical protein, conserved	687	**PVX_096080**	mago nashi domain containing protein	147	**PVX_003735**	DNA repair endonuclease, putative,RAD2 endonuclease, putative	843
**PVX_093495**	hypothetical protein, conserved	693	**PVX_081780**	hypothetical protein, conserved	162	**PVX_096085**	hypothetical protein, conserved	1051	**PVX_003740**	cysteine desulfuration protein SufE, putative (SufE)	241
**PVX_093500**	hypothetical protein, conserved	93	**PVX_081785**	hypothetical protein, conserved	696	**PVX_096090**	exonuclease I, putative	516	**PVX_003745**	pantothenate transporter, putative (PAT)	532
**PVX_093505**	hypothetical protein, conserved	396	**PVX_081790**	hypothetical protein, conserved	96	**PVX_096095**	kinesin-19, putative	1222	**PVX_003750**	pentafunctional AROM polypeptide, putative (AROM)	1225
**PVX_093510**	alpha/beta hydrolase	239	**PVX_081792**	hypothetical protein	4762	**PVX_096105**	kelch domain-containing protein	357	**PVX_003755**	hypothetical protein, conserved	518
**PVX_093515**	GTPase activator	337	**PVX_081795**	hypothetical protein, conserved	108	**PVX_096110**	hypothetical protein, conserved	1987	**PVX_003760**	transcription factor, putative	105
**PVX_093520**	peptidyl-prolyl cis-trans isomerase	188	**PVX_081800**	hypothetical protein, conserved	632	**PVX_096115**	protein kinase, putative	1056	**PVX_003765**	adenylosuccinate lyase (ASL)	459
**PVX_093525**	hypothetical protein, conserved	126	**PVX_081805**	AP-4 complex subunit beta, putative	756	**PVX_096120**	hypothetical protein, conserved	296	**PVX_003770**	merozoite surface protein 5	119
**PVX_093530**	pseudouridylate synthase	359	**PVX_081810**	transcription factor with AP2 domain(s), putative (AP2-L)	743	**PVX_096125**	mitochondrial import inner membrane translocase subunit TIM14, putative (PAM18)	114	**PVX_003775**	merozoite surface protein 4, putative	103
**PVX_093535**	hypothetical protein, conserved	2593	**PVX_081815**	hypothetical protein, conserved	157	**PVX_096130**	3-demethylubiquinone-9 3-methyltransferase, putative	279	**PVX_003780**	hypothetical protein, conserved	908
**PVX_093540**	methionine aminopeptidase	494	**PVX_081820**	hypothetical protein, conserved	271	**PVX_096135**	type 2A phosphatase-associated protein 42, putative (TAP42)	313	**PVX_003785**	iron-sulfur cluster assembly accessory protein, putative	111
**PVX_093545**	hypothetical protein, conserved	277	**PVX_081825**	hypothetical protein	231	**PVX_096140**	hypothetical protein, conserved	58	**PVX_003855**	hypothetical protein, conserved	1076
**PVX_093550**	cactin homolog	494	**PVX_081830**	Plasmodium exported protein, unknown function	456	**PVX_096145**	Rab GTPase activator and protein kinase, putative	742	**PVX_003860**	KRR1 small subunit processome component, putative (KRR1)	285
**PVX_093555**	20S proteasome beta subunit	255	**PVX_081832**	Plasmodium exported protein, unknown function	99	**PVX_096150**	hypothetical protein, conserved	719	**PVX_003865**	hypothetical protein, conserved	915
**PVX_093560**	tubulin gamma chain (g-tub)	451	**PVX_096950**	tryptophan-rich antigen (Pv-fam-a)	282	**PVX_096155**	hypothetical protein, conserved	1089	**PVX_003870**	hypothetical protein, conserved	871
**PVX_093565**	hypothetical protein, conserved	437	**PVX_096955**	Plasmodium exported protein, unknown function	188	**PVX_096165**	metallo-hydrolase/oxidoreductase, putative	487	**PVX_003880**	acyl carrier protein, putative (ACP)	119
**PVX_093570**	AAA family ATPase	695	**PVX_096995**	tryptophan-rich antigen (Pv-fam-a)	268	**PVX_096170**	hypothetical protein, conserved	109	**PVX_003885**	ribosome-recycling factor, putative (RRF1)	222
**PVX_093575**	DNA repair protein rad54	636	**PVX_097005**	Plasmodium exported protein, unknown function	141	**PVX_096175**	dynactin subunit 5, putative	203	**PVX_003890**	hypothetical protein, conserved	221
**PVX_093580**	mitogen-activated protein kinase organizer 1	356	**PVX_097010**	hypothetical protein	88	**PVX_096180**	hypothetical protein	625	**PVX_003895**	hypothetical protein, conserved	207
**PVX_093585**	SF-assemblin	378		**total**	**16240**	**PVX_096185**	hypothetical protein, conserved	291	**PVX_003900**	6-cysteine protein	1928
**PVX_093590**	hypothetical protein, conserved	513				**PVX_096195**	hypothetical protein, conserved	346	**PVX_003905**	6-cysteine protein	2273
**PVX_093595**	peptidyl-prolyl cis-trans isomerase	394				**PVX_096200**	hypothetical protein, conserved	303	**PVX_003910**	phospholipase A2, putative	635
**PVX_093600**	hypothetical protein, conserved	120				**PVX_096205**	hypothetical protein, conserved	237	**PVX_003915**	hypothetical protein, conserved	267
**PVX_093605**	protein phosphatase	461				**PVX_096210**	hypothetical protein, conserved	273	**PVX_003920**	2C-methyl-D-erythritol 2,4-cyclodiphosphate synthase, putative	192

ID_chr1	gene	No of aa position used for the phylogeny
**PVX_093607**	hypothetical protein, conserved	202
**PVX_093610**	adenylyl cyclase beta	1177
**PVX_093615**	inositol phosphatase	897
**PVX_093620**	hypothetical protein, conserved	387
**PVX_093625**	hypothetical protein, conserved	914
**PVX_093630**	1-cys peroxiredoxin (1-cyspxn)	219
**PVX_093635**	transcription factor with AP2 domain(s)	503
**PVX_093640**	glutamate dehydrogenase	1022
**PVX_093645**	hypothetical protein, conserved	1066
**PVX_093650**	mannose-6-phosphate isomerase	617
**PVX_093655**	sentrin-specific protease 2	812
**PVX_093660**	hypothetical protein, conserved	123
**PVX_093665**	hypothetical protein, conserved	523
**PVX_093670**	protein kinase	493
**PVX_093675**	von willebrand factor a-domain-related protein (WARP)	264
**PVX_093680**	Phist protein (Pf-fam-b)	183
	**total**	**9402**

ID_chr2	gene	No of aa position used for the phylogeny

ID_chr3	gene	No of aa position used for the phylogeny
**PVX_096215**	hypothetical protein, conserved	416
**PVX_096220**	hypothetical protein, conserved	78
**PVX_096225**	hypothetical protein, conserved	450
**PVX_096230**	hypothetical protein, conserved	211
**PVX_096235**	GTP-binding protein, putative	391
**PVX_096240**	hypothetical protein, conserved	531
**PVX_096245**	rhoptry-associated leucine zipper-like protein 1, putative (RALP1)	281
**PVX_096250**	PelOta protein homologue, putative	352
**PVX_096253**	conserved Plasmodium protein, unknown function	77
**PVX_096255**	hypothetical protein, conserved	103
**PVX_096260**	hypothetical protein, conserved	455
**PVX_096265**	40S ribosomal protein S5, putative	194
**PVX_096271**	conserved Plasmodium protein, unknown function	219
**PVX_096273**	DEAD/DEAH box ATP-dependent RNA helicase, putative	578
**PVX_096275**	hypothetical protein, conserved	590
**PVX_096280**	hypothetical protein, conserved	219
**PVX_096285**	hypothetical protein, conserved	1685
**PVX_096289**	hypothetical protein	167
**PVX_096292**	HAM1 domain containing protein	197
**PVX_096295**	hypothetical protein, conserved	1359
**PVX_096300**	hypothetical protein, conserved	225
**PVX_096302**	conserved Plasmodium protein, unknown function	133
**PVX_096305**	ferrodoxin reductase, putative	599
**PVX_096307**	conserved Plasmodium protein, unknown function	104
**PVX_096320**	exosome complex component CSL4, putative (CSL4)	188
**PVX_096325**	hypothetical protein, conserved	1493
**PVX_096330**	hypothetical protein, conserved	384
**PVX_096335**	40S ribosomal protein S10, putative	132
**PVX_096340**	60S ribosomal protein L11, putative	172
**PVX_096345**	hypothetical protein, conserved	311
**PVX_096350**	hypothetical protein, conserved	570
**PVX_096355**	actin-related protein, putative (ARP6)	588
**PVX_096360**	serine/threonine-protein kinase NEK4, putative	309
**PVX_096365**	hypothetical protein	176
**PVX_096370**	hypothetical protein, conserved	131
**PVX_096380**	conserved Plasmodium protein, unknown function	247
**PVX_096385**	hypothetical protein, conserved	272
**PVX_096395**	hypothetical protein, conserved	544
**PVX_096400**	prefoldin subunit 3, putative	188
**PVX_096405**	30S ribosomal protein S8, putative	128
**PVX_096410**	cysteine repeat modular protein 2, putative (CRMP2)	2428
	**total**	**17875**

ID_chr4	gene	No of aa position used for the phylogeny
**PVX_003925**	hypothetical protein, conserved	447
**PVX_003930**	hypothetical protein	325
**PVX_003935**	amine transporter, putative	925
**PVX_003940**	RING zinc finger protein, putative	338
**PVX_003945**	ATP-dependent RNA helicase UAP56, putative (UAP56)	460
**PVX_003950**	transport protein SEC61 gamma subunit, putative	81
**PVX_003955**	60S ribosomal protein L37a, putative	95
**PVX_003960**	hypothetical protein, conserved	1539
**PVX_003965**	transporter, putative	455
**PVX_003970**	50S ribosomal protein L33, putative	118
**PVX_003975**	hypothetical protein, conserved	848
**PVX_003980**	hypothetical protein, conserved	445
**PVX_003985**	syntaxin, putative	304
**PVX_003990**	hypothetical protein, conserved	354
**PVX_003995**	hypothetical protein, conserved	286
	**total**	**7020**

Table S5 Model test for phylogeny using genome encoded gene sequences of seven *Plasmodium* species

Model	−Ln	No of parameters				AIC	Bootstrap
		branch(2n−3)	+F	+G	total		
GTR+Γ	−1956269.6	11	208	1	220	**3,912,979**	all 100
BLOSUM62+Γ	−2052737.1	11	0	1	12	4,105,498	all 100
CPREV+Γ	−2034964.4	11	0	1	12	4,069,953	all 100
JTT+Γ	−2008632.9	11	0	1	12	4,017,290	all 100
LG+Γ	−2021124.7	11	0	1	12	4,042,273	all 100
VT+Γ	−2031452.3	11	0	1	12	4,062,929	all 100
WAG+Γ	−2034598.8	11	0	1	12	4,069,222	all 100
BLOSUM62+Γ+F	−2029042.9	11	19	1	31	4,058,148	all 100
CPREV+Γ+F	−2010443.7	11	19	1	31	4,020,949	all 100
JTT+Γ+F	−1977359.6	11	19	1	31	3,954,781	all 100
LG+Γ+F	−1997772.3	11	19	1	31	3,995,607	all 100
VT+Γ+F	−2005338.9	11	19	1	31	4,010,740	all 100
WAG+Γ+F	−2001293.2	11	19	1	31	4,002,648	all 100

Table S6 Approximately unbiased tese of 15 Ttree topologies

MODEL	GTR	BLOSUM62		CPREV		JTT		LG		VT		WAG	
Tree		+Γ	+Γ+F	+Γ	+Γ+F	+Γ	+Γ+F	+Γ	+Γ+F	+Γ	+Γ+F	+Γ	+Γ+F
(go,(vi,(cy,(in,(fr,(kn,co))))))	0.9980	0.9960	0.9950	1.0000	0.9980	0.9980	0.9980	0.9970	0.9950	0.9970	0.9970	0.9970	0.9960
(go,(vi,((cy,in),(fr,(kn,co)))))	0.0020	0.0004	0.0001	0.0010	0.0010	0.0020	0.0002	0.0003	0.0003	0.0010	0.0001	0.0010	0.0001
(go,(vi,((cy,(fr,(kn,co))),in)))	0.0030	0.0060	0.0060	0.0010	0.0040	0.0040	0.0040	0.0060	0.0080	0.0040	0.0050	0.0060	0.0060
(go,(cy,(vi,(in,(fr,(kn,co))))))	0.0000	0.0000	0.0000	0.0000	0.0000	0.0001	0.0001	0.0000	0.0000	0.0000	0.0000	0.0000	0.0000
(go,(cy,(in,(vi,(fr,(kn,co))))))	0.0000	0.0000	0.0000	0.0000	0.0001	0.0010	0.0000	0.0000	0.0004	0.0000	0.0000	0.0000	0.0002
(go,(cy,((fr,(kn,co)),(vi,in))))	0.0000	0.0000	0.0000	0.0000	0.0020	0.0010	0.0000	0.0000	0.0000	0.0000	0.0000	0.0000	0.0000
(go,(in,(vi,(cy,(fr,(kn,co))))))	0.0000	0.0000	0.0000	0.0010	0.0003	0.0002	0.0000	0.0000	0.0001	0.0000	0.0000	0.0000	0.0002
(go,(in,((vi,cy),(fr,(kn,co)))))	0.0001	0.0000	0.0000	0.0004	0.0000	0.0000	0.0000	0.0000	0.0000	0.0000	0.0000	0.0000	0.0000
(go,(in,((vi,(fr,(kn,co))),cy)))	0.0000	0.0000	0.0000	0.0000	0.0000	0.0080	0.0000	0.0001	0.0000	0.0000	0.0000	0.0000	0.0000
(go,((fr,(kn,co)),(vi,(cy,in))))	0.0210	0.0000	0.0000	0.0000	0.0000	0.0001	0.0000	0.0020	0.0000	0.0002	0.0000	0.0000	0.0090
(go,((fr,(kn,co)),((vi,cy),in)))	0.0000	0.0000	0.0000	0.0003	0.0000	0.0000	0.0000	0.0000	0.0000	0.0001	0.0000	0.0000	0.0000
(go,((fr,(kn,co)),((vi,in),cy)))	0.0000	0.0000	0.0000	0.0002	0.0010	0.0001	0.0000	0.0004	0.0001	0.0000	0.0000	0.0000	0.0000
(go,((vi,cy),(in,(fr,(kn,co)))))	0.0000	0.0000	0.0000	0.0000	0.0000	0.0000	0.0000	0.0130	0.0000	0.0000	0.0000	0.0000	0.0000
(go,((vi,in),(cy,(fr,(kn,co)))))	0.0000	0.0000	0.0000	0.0030	0.0002	0.0020	0.0000	0.0000	0.0000	0.0000	0.0004	0.0000	0.0000
(go,((vi,(fr,(kn,co))),(cy,in)))	0.0000	0.0000	0.0000	0.0000	0.0000	0.0000	0.0000	0.0000	0.0000	0.0000	0.0000	0.0000	0.0000
